# The association between different sources of distraction and symptoms of attention deficit hyperactivity disorder

**DOI:** 10.3389/fpsyt.2023.1173989

**Published:** 2023-07-27

**Authors:** Jahla B. Osborne, Han Zhang, Madison Carlson, Priti Shah, John Jonides

**Affiliations:** Department of Psychology, University of Michigan, Ann Arbor, MI, United States

**Keywords:** attention-deficit/hyperactivity disorder, mind-wandering, distraction, network analysis, dominance analysis

## Abstract

**Introduction:**

Adults with Attention-Deficit/Hyperactivity Disorder (ADHD) are generally distractible. Yet, the precise relationship between ADHD and distractibility remains under-specified in two respects. First, different sources of distraction, such as background noise or mind wandering, may not be equally associated with ADHD. Second, ADHD itself comprises a variety of symptoms that show considerable heterogeneity and it is unclear which ADHD symptoms are associated with which type of distraction.

**Methods:**

The current study addresses these questions using one clinically evaluated sample (*N* = 69) and two large non-clinically evaluated samples (*N* = 569, *N* = 651). In all samples, participants completed questionnaires about their susceptibility to external distraction, unwanted intrusive thoughts, spontaneous mind-wandering and ADHD symptomatology.

**Results:**

Traditional regression and novel network analyses revealed an overwhelming contribution of spontaneous mind-wandering in explaining ADHD symptoms, although external distraction and unwanted intrusive thoughts were also associated with a small number of ADHD symptoms.

**Discussion:**

Findings support a growing body of literature linking spontaneous mind-wandering and ADHD, and they highlight the heterogeneity in the association between ADHD symptoms and different sources of distraction.

## 1. Introduction

Attention-Deficit/Hyperactivity Disorder (ADHD) is a neurodevelopmental disorder that encompasses inattentive, hyperactive and impulsive behavior (1). These symptoms are thought to fall on a continuum of severity, with a formal diagnosis of ADHD reflecting those at the high end of the spectrum (2). Although typically diagnosed in children, ADHD often continues into adulthood and is estimated to affect 4.4% of adults in the United States and 5.3% of adults worldwide (3, 4). The hyperactive symptoms are thought to improve into adulthood, but the inattentive symptoms tend to persist, which can lead to serious functional consequences such as lower college completion rates, higher job-change rates and higher incarceration rates (5–9). In that the inattentive symptoms continue to be problematic, knowing more about how distraction manifests in adults with ADHD is important for understanding the underlying mechanisms and for improving the quality of life.

Individuals with a high level of ADHD symptomatology are often generally described as being more distractible (1). Yet, the precise nature of distractibility remains under-specified in two respects. First, distraction can manifest in a variety of forms; one might be distracted by background noise in a cafe (external distraction), daydreams about next weekend’s plans (internal distraction through mind-wandering), or ruminations about a recent awkward social interaction (internal distraction through unwanted intrusive thoughts). Are different sources of distractions equally associated with ADHD? Second, ADHD consists of a set of symptoms that characterize attention deficits from different aspects, and these symptoms may be differentially related to distraction. Given the heterogeneity in both distraction types and ADHD symptoms, it is necessary to simultaneously measure multiple distraction types and to use analytical approaches that could uncover differential relations at the symptom level. The current study focuses on three types of distraction, spontaneous mind-wandering, external distraction and unwanted intrusive thoughts, and their potentially different associations with individual ADHD symptoms.

### 1.1. Distraction types and ADHD

While the word “distraction” can be used to refer to both external and internal sources, the study of distraction has traditionally focused on external stimulation. As a result, many studies of distractibility in ADHD have focused on this particular type of distraction. Indeed, “being easily distracted by extraneous stimuli” is a common complaint by people with a high level of ADHD symptomatology, and external distractibility has long been considered as one of the diagnostic criteria of ADHD (10). Studies using task-based measures have also found that task-irrelevant distractors (e.g., unrelated auditory or visual stimuli) disrupt performance more in ADHD participants than in healthy controls (11–14). Overall, previous research shows a solid connection between external distractibility and ADHD.

External stimulation, however, is not the only source of distraction. Studies have shown that performance can be impaired by internal sources of information in the absence of external distraction (15, 16). In particular, mind-wandering refers to a collection of mental phenomena that entail a shift of attention away from a task toward “unrelated inner thoughts, fantasies, feelings and other musings” (17). For example, while trying to listen to a course lecture, a person might think about plans for an upcoming social event. Mind-wandering may occur spontaneously or deliberately (18). A growing body of literature provides evidence that individuals with a higher level of ADHD symptomatology experience more frequent episodes of spontaneous mind-wandering but not deliberate mind-wandering (15, 18–20). These results are consistent with the emerging perspective that spontaneous mind wandering is a core feature of ADHD (18).

A similar albeit not identical form of internal distraction is unwanted intrusive thoughts. As the name suggests, these thoughts are often negative, intrusive and difficult to control (e.g., keep worrying about an upcoming exam) (21–23). Like mind-wandering, unwanted intrusive thoughts also capture mental capacity, and, as a result, are associated with impaired performance (24). Different from mind-wandering, however, unwanted intrusive thoughts typically involve negative mental content that may occur repeatedly (e.g., keep thinking about an unpleasant idea) and they are often associated with conscious appraisals and attempts to resist (25). A recent study by Zhang et al. (26) showed that unwanted intrusive thoughts and spontaneous mind-wandering were correlated at *r* = 0.62 ~ 0.68 at the latent level, indicating that they are indeed highly related yet distinct forms of distraction. Furthermore, a handful of studies have found that unwanted intrusive thoughts are correlated with inattentive-ADHD symptoms but not with hyperactive/impulsive-ADHD symptoms (16, 27). Thus, it appears that not all ADHD symptoms are equally associated with unwanted intrusive thoughts.

Susceptibility to external and internal distractions in ADHD may be based on different neural circuits that are involved in filtering different types of distraction. Bottom-up attentional processing involves the automatic allocation of attention to sensory information in the environment (28). To avoid being externally distracted and successfully carry-out goal directed activities, individuals must filter out irrelevant stimuli in their environment. On the other hand, top–down attentional processing can be described as attentional capture that is voluntarily selected based on prior experiences, current goals and motivation (28). The underlying neural circuits associated with ADHD are thought to include a hyperactive bottom-up attentional processing system and a hypoactive top-down attentional processing system (13, 29). Research suggests bottom-up processing systems within individuals with ADHD may be overly sensitive (13, 30). In other words, individuals with ADHD may engage in enhanced processing of both relevant and irrelevant external stimuli, manifesting as an increased susceptibility to external distraction. Increased susceptibility to internal forms of distraction (mind-wandering, unwanted intrusive thoughts) may suggest issues within top-down processing circuits, which in turn may make it difficult for individuals with ADHD to exert cognitive control to focus on the task at hand rather than on internal distracting thoughts. It is well documented in the cognitive control literature that individuals with ADHD struggle with employing mechanisms for top-down attentional control and subsequently tend to perform worse on these types of tasks compared to healthy controls (11–13). Overall, previous research indicates that external distraction, spontaneous mind-wandering and unwanted intrusive thoughts are all somewhat associated with ADHD symptomatology. However, because the aforementioned studies did not measure the three types of distraction simultaneously, it remains unclear which type of distraction has the strongest relationship. Additionally, these studies did not analyze ADHD at the symptom level, thus ignoring the potential heterogeneity of their relationships with different types of distractions. We shall elaborate on this issue next.

### 1.2. The prevalence of a common factor approach in studying ADHD

Most studies of external distraction, mind-wandering and unwanted intrusive thoughts have focused on explaining ADHD measured by aggregate scores. Although some studies have examined heterogeneity at the ADHD subtype level (Inattentive-ADHD, Hyperactive/Impulsive-ADHD, Combined-ADHD) generally, these studies measure ADHD by summing the number of symptoms present, averaging across symptom ratings, or simply comparing individuals with a clinical diagnosis versus healthy controls (11–13, 31). By aggregating across symptoms, researchers study the aspect of ADHD that is defined as the commonality across these symptoms whereas the potential heterogeneity among individual symptoms may be lost. For example, each symptom of ADHD could be equally related to external distraction, spontaneous mind-wandering and unwanted intrusive thoughts. Alternatively, some ADHD symptoms may be more associated with a specific type of distraction or not related to distraction at all.

For example, Zhang et al. (26), used a latent factor approach and showed that a substantial portion of variance in ADHD symptomatology (as indicated by *Adult ADHD Self Report Scale (ASRS)* scores) can be accounted for by a “general distractibility factor” (*d*-factor) that is composed of external distraction, unwanted intrusive thoughts, and spontaneous mind-wandering. This seems to suggest that ADHD symptoms can best be described as a susceptibility to general distraction. However, this result was derived from examining the link between two general constructs, general distractibility and ADHD symptomatology, by extracting the common variance shared across different types of distraction and the common variance shared across different ADHD symptoms. As such, it remains unclear if different symptoms of ADHD may be related to different forms of distraction. Consider the following scenarios: Say the symptom *often forgetful* has a strong association to spontaneous mind-wandering, but *often fidgets* has only a moderate association to external distraction. By contrast, imagine that both *often forgetful* and *often fidgets* are equally strongly related to both spontaneous mind-wandering and external distraction. When using a latent factor approach, these symptoms would be summed together to extract ADHD as a whole. In doing so, the nuance inherent in individual symptoms is lost. That is, we are unable to distinguish if symptoms are equally or differentially related to distractions, because in both cases the overall relationship between the two aggregate scores (distractibility and ADHD symptomatology) will be high.

There is a well-documented body of research by Fried and Colleagues about Major Depressive Disorder (MDD) which serves as a good analogy. Their studies uncovered that individual depressive symptoms were differently associated with functional impairment (32) versus depressive risk factors (33). This highlights that sum-scoring depressive symptoms can be problematic in that different patients might have similar summed scores of depressive symptoms, but different profiles of symptomatology. For example, Lux and Kendler (34) examined the association between MDD symptoms and depression risk factors in a sample of twins. When depression symptoms were sum-scored together the results indicated that female participants were at increased odds of developing depression. However, analysis of individual symptoms indicated that male participants were at increased odds of experiencing *suicidal ideation*, while female participants were at increased odds of experiencing issues in *focusing*, *sleeping*, and *eating* to name a few. These findings highlight the importance of examining disorders at the individual-symptom level.

It is well documented in the literature that ADHD symptoms are heterogeneous in nature (35, 36). For example, separate individuals can both meet diagnostic criteria for ADHD, but also report vastly different combinations of symptoms, report differing levels of functional impairment, as well as different comorbid disorders (if at all). To better understand the structure of ADHD, the traditional approach has been to divide the 18 symptoms into dimensions (inattentive, hyperactive/impulsive). However, even within dimensions there is heterogeneity. For example, Zoromski et al. (37) examined if individual symptoms are more closely associated with social, academic or behavioral impairments through stepwise multiple regression. Out of the nine inattentive symptoms, they found *poor follow through, task avoidance* and *difficulty organizing* to be significantly associated with academic impairment in adolescents according to teacher ratings. By contrast, in early childhood they found other inattentive symptoms such as *forgetful* and *careless mistakes* to be significantly associated with academic impairment according to teacher ratings. These results highlight heterogeneity within symptom dimensions in relation to impairment, as well as changes across development. Other studies assessing ADHD from the individual symptom level have found certain symptoms increase the odds of a clinical diagnosis, such as *careless mistakes*, *difficulty sustaining attention* and *physically restless* to name a few (38). Overall, the past literature suggests that the presentation of ADHD differs from person to person; given this heterogeneity it is necessary to study ADHD at the individual symptom level.

For the current study, we aim to identify the associations between individual ADHD symptoms and different types of distraction (external distraction, spontaneous mind-wandering and unwanted intrusive thoughts). We intend to better understand the diversity of the relationships between ADHD and distractibility. Let us first have a discussion of the complementary methods used to carry out this symptom analysis.

### 1.3. Methods to examine the ADHD-distraction relationship at the symptom level

#### 1.3.1. Dominance analysis

To assess the relative importance of each distraction type in explaining individual symptoms, we will conduct a dominance analysis. While a traditional multiple regression analysis allows one to assess the unique predictive strength of a predictor while controlling for other predictors, it does not *compare* the predictive strength among the predictors. That is, it does not allow one to assess the relative importance of a predictor. This drawback, however, can be easily overcome by a dominance analysis. A dominance analysis examines the dominance of a predictor over another predictor by comparing their incremental R^2^ contributions across all subset models (39). The most restricted form of dominance is called *complete dominance*, in which a predictor makes a larger contribution compared to another predictor in all subset models (40, 41). For example, in a three-predictor model, X1 is said to have complete dominance over X2 if the incremental R^2^ contribution of X1 is larger than that of X2 in all subset models, that is, in the null model and in the model consisting of X3. Dominance analysis is an advantageous approach to answering our research question in that it allows us to identify if there is a source of distraction that is relatively more important in predicting individual symptoms of ADHD compared to other forms of distraction. For example, to the extent that spontaneous mind-wandering is a core feature of ADHD, one would expect spontaneous mind-wandering to be the dominant predictor over external distraction and unwanted intrusive thoughts in explaining most of the ADHD symptoms.

#### 1.3.2. Network analysis

Network analysis has become an increasingly popular approach for understanding psychiatric disorders. A *psychological network* is a visual and statistical model of how psychological variables interact among one another (42). In a psychological network, observed variables, such as psychopathology symptoms, are represented as *nodes* (visualized with circles) with their relationships being represented by *edges* (visualized with lines that connect the circles). The strength of the connection, or *edge weight*, may differ, with thicker lines indicating stronger relationships and thinner lines indicating weaker relationships. Edge weights may also be positive (visualized with blue lines) or negative (visualized with red lines). In a psychological network, the edges usually represent partial correlations, that is, the association between two nodes given all other nodes in the network. Compared to the dominance analysis approach, a clear advantage of the network approach is that it can simultaneously estimate relationships among multiple variables of interest. Thus, two nodes that are connected with each other indicate unique associations between the two variables that cannot be explained by other variables in the network. Conversely, two nodes that are not connected with each other (i.e., edge weight = 0) in the network are conditionally independent, meaning that their relationship can be accounted for by other variables in the network. The ability to model conditional independencies makes network analysis useful for exploring potential causal mechanisms (43). Specifically, we will use regularized partial correlations in our network in that regularization eliminates partial correlations of zero (no association) from the network model (44). Ultimately, this removes any potential spurious relationships between nodes and allows for a more interpretable network (44).

More fundamentally, the network approach provides a new way to conceptualize the relationship between variables. Instead of assuming that observed variables are correlated because of a common underlying cause, the network approach explains the correlated nature *via* the direct relationships among the observed variables (45, 46). For example, someone who is susceptible to spontaneous mind-wandering also has difficulties keeping attention when doing boring, repetitive work (*ASRS* item 8), and someone who has difficulties keeping attention when doing boring, repetitive work also makes careless mistakes (*ASRS* item 7). On the other hand, the same person who is susceptible to spontaneous mind-wandering may not tend to have difficulties unwinding and relaxing themselves (*ASRS* item 14), but this symptom may be instead associated with other variables in the network. In addition to the observation of individual edges, several well-established metrics are available to quantify the overall importance of a node in the network (42). Overall, network analysis provides a nuanced look into the pathways in which vulnerabilities to different types of distraction might interact with individual ADHD symptoms.

A growing body of literature has emerged in recent years that centers around understanding the network structure of the 18 ADHD symptoms that are included in clinical diagnosis (47–49). These studies have generally focused on establishing “core” features of ADHD, as well as detecting clusters of symptoms. The present study differs from the existing literature in that we are interested in how individual ADHD symptoms are associated with external distraction, spontaneous mind-wandering and unwanted intrusive thoughts in the network.

### 1.4. Present study

The goal of the present study is to characterize the association between individual ADHD symptoms and three types of distractions (external distraction, spontaneous mind-wandering and unwanted intrusive thoughts). We examined one clinically evaluated sample (N = 69) and two large non-clinically evaluated cohorts (N = 569, N = 651) in which participants self-reported their susceptibility to external distraction, spontaneous mind-wandering and unwanted intrusive thoughts using multiple questionnaires to define each distraction construct. Additionally, we measured ADHD symptoms using the *Structured Clinical Interview for DSM-5 (Research Version) (SCID-5-RV)* in the clinically evaluated sample, and the *Adult ADHD Self Report Scale (ASRS)* in the non-clinically evaluated samples. To analyze our data, we implemented a rigorous methodological approach where we used a combination of dominance analysis and network analysis to identify any symptoms that are not equally associated with each type of distraction, but rather are more related to a specific type of distraction.

## 2. Materials and methods

### 2.1. Participants

#### 2.1.1. Prolific sample

Participants were recruited from the online research system, *Prolific.co*. All participants were native English speakers, between the age of 18–35, living in the U.S. or Canada, and completed at least 50 previous *Prolific.co* submissions at a 95% approval rating. Participants were removed if they failed 2 (of 4) attention checks. Participants were compensated $3.67 for completing distraction and ADHD symptomatology online questionnaires that took an average of 24 min. One participant was excluded from data analysis for a duplicated IP address. The final sample consisted of 651 participants. The mean age was 26.9 with a SD of 4.98. The sample was 45.31% female and 67.7% Caucasian. This dataset originates from Zhang et al. (26).

#### 2.1.2. University sample

Six hundred and fifteen participants were recruited from the University of Michigan Psychology Subject Pool. Participants received 0.5 h of subject pool credit for completing online questionnaires about distraction and ADHD symptomatology. Forty-six participants were excluded from data analysis for failing 2 (of 4) attention checks. Therefore, the final sample consisted of 569 individuals. The mean age was 18.83 with a SD of 1.15. The sample was 66.26% female and 56.8% Caucasian. This dataset originates from Zhang et al. (26).

#### 2.1.3. Clinically evaluated sample

We used a prescreening survey of the entire University of Michigan Psychology Subject Pool to identify participants who self-reported a clinical diagnosis of ADHD. We enrolled 69 individuals. All participants received 1 h of subject pool credit for completing a formal clinical interview using the ADHD module (Module-K) of the *Structured Clinical Interview for DSM-5 (Research Version)(SCID-5-RV)* and filling out 15 online questionnaires regarding distraction and ADHD symptomatology (see below for details). Additionally, two participants indicated a comorbid diagnosis of depression (with ADHD). In that ADHD and MDD are highly comorbid, both of these participants were retained (3). The mean age was 18.96 with a SD of 2.00. The sample was 50.7% male and 63.8% Caucasian. Previous clinical ADHD studies examining cognitive processes report similar sample sizes compared to our current sample (50, 51).

### 2.2. Measures

#### 2.2.1. ADHD diagnostic measure

##### 2.2.1.1. Module K: structured clinical interview for DSM-5-research version

Module K of the *SCID-5-RV* is structured to assess and diagnose adults with ADHD and was administered by formally trained graduate students and research assistants. The module is made up of 5 major criteria. Criterion A assesses inattentive (questions 1–9) and hyperactive–impulsive (questions 10–18) symptoms. For example, “...have you often missed important details or made mistakes at work (or school) or while taking care of things at home?” Each item is rated on a 3-point scale, ranging from 1 “absent or false” to 3 “threshold or true.” This module uses a dichotomous scoring method that indexes whether a symptom is present or not. Only responses of “threshold or true” are coded as symptom present, while responses of “subthreshold” or “absent or false” are coded as symptom absent. To move on to subsequent sections, a participant must endorse at least 5 inattentive symptoms or at least 5 hyperactive–impulsive symptoms. Criterion B assesses when symptoms first emerged; they must have been present by 12 years of age. Criterion C assesses where symptoms emerge; to continue the survey, participants must endorse symptoms appearing in two or more areas of their life (e.g., school, work, home). Criterion D examines severity of symptoms; participants must endorse that their symptoms seriously impair functioning in everyday settings (e.g., work, school, social settings). Finally, Criterion E investigates if a separate psychotic disorder is present. This section aims to ensure that the endorsed ADHD symptoms are not better described by psychotic disorder(s) (if any present). Participants are considered to have a clinical diagnosis of ADHD if they fit Criteria A-E. In that our research question seeks to understand the individual symptoms of ADHD, our analyses consider their dichotomized scores of each individual symptom (symptom absent = 0, symptom present = 1) from this diagnostic measure (52).

#### 2.2.2. ADHD symptomatology measure

##### 2.2.2.1. Adult ADHD self-report scale

The *ASRS* assesses self-reported ADHD symptoms. This 18-item scale is based on the 18 adult ADHD symptoms outlined in the Diagnostic and Statistical Manual of Mental Disorders Fourth Edition (DSM-IV-TR). Each question is rated on a 5-point scale, ranging from 0 “Never” to 4 “Very often.” In that our research question seeks to understand the individual symptoms of ADHD, these continuously scored individual items were not summed together (53).

#### 2.2.3. Mind-wandering scales

##### 2.2.3.1. Imaginal processes inventory–mind-wandering

The *IPI-MW* measures one’s tendency to experience task-unrelated thoughts. The inventory consists of 6 items, for example “Even when I am listening to an interesting speaker, my mind wanders.” Each item is rated on a 5-point scale from 0 “definitely not true for me” to 4 “very true for me.” The final score is generated by summing the scores of all items (54, 55).

##### 2.2.3.2. Mind-wandering–spontaneous

The *MW-S* measures one’s propensity to spontaneously mind-wander. The questionnaire consists of 4 items, for example “I mind-wander even when I’m supposed to be doing something else.” Each item is rated on a 7-point scale from 1 “rarely” to 7 “a lot.” However, the third item “It feels like I do not have control over when my mind wanders,” is rated from 1 “almost never” to 7 “almost always.” The final score is generated by summing the scores of all items (56).

##### 2.2.3.3. Daydreaming frequency scale

The *DDFS* measures how often an individual daydreams. The scale consists of 12 items, for example “I daydream ____.” Participants then fill in the blank based on a 5-point scale ranging from 1 “infrequently” to 5 “many different times during the day.” However, some items incorporate a different range of responses. For example, item 5, asks “When I am ***not*** paying close attention to my job, a book, or TV, I tend to be daydreaming ______,” and the responses range from 1 “0% of the time” to 5 “75% of the time.” All in all, every item is scored on a 5-point scale even when the provided responses differ between questions. The final score is generated by summing the scores of all items (55).

#### 2.2.4. External distraction scales

##### 2.2.4.1. Attentional control–distraction

The *AC-D* examines one’s tendency to be externally distracted. The questionnaire consists of 4 items, for example “It is very hard for me to concentrate on a difficult task when there are noises around.” Each item is rated on a 5-point scale from 1 “Almost never” to 5 “Always.” The final score is generated by summing the scores of all items (56).

##### 2.2.4.2. Imaginal processes inventory–distractibility

The *IPI-D* measures propensity to be distractible during a task when competing stimuli are also present. The inventory consists of 5 items, for example “I find it hard to read when someone is on the telephone in a neighboring room.” Each item is rated on a 5-point scale from 0 “definitely not true for me” to 4 “very true for me.” The final score is generated by summing the scores of all items (54, 55).

##### 2.2.4.3. Attentional style questionnaire–external

In general, the *ASQ* assesses one’s capability to sustain attention on target stimuli, while avoiding being distracted by irrelevant stimuli. Specifically, the *ASQ-E* is the 5-item subscale that focuses on tendency to be externally distracted, for example “I am often the first one to notice something has changed in a room.” Each item is rated on a 6-point scale from 1 “in total disagreement” to 6 “in total agreement” scale. The final score is generated by summing the scores of all items. All 17 items from the *ASQ* were presented, but only the 5-item *ASQ-E* subscale data were analyzed in this study (57).

#### 2.2.5. Unwanted intrusive thought scales

##### 2.2.5.1. Perseverative thinking questionnaire

The *PTQ* examines one’s tendency to experience repetitive negative thoughts. The questionnaire consists of 15 items, for example “I think about many problems without solving any of them.” Each item is rated on a 5-point scale from 0 “never” to 4 “almost always.” The final score is generated by summing the scores of all items (21).

##### 2.2.5.2. White bear suppression inventory

The *WBSI* measures one’s tendency to suppress unwanted thoughts. The inventory consists of 15 items, for example “Sometimes I really wish I could stop thinking.” Each item is rated on a 5-point scale from 1 “strongly disagree” to 5 “strongly agree.” The final score is generated by summing the scores of all items (58).

##### 2.2.5.3. Penn state worry questionnaire

The *PSWQ* measures one’s susceptibility to excessive worry. The questionnaire consists of 16 items, for example “As soon as I finish one task, I start to worry about everything else I have to do.” Each item is rated on a 5-point scale from 1 “not at all typical of me” to 5 “very typical of me.” The final score is generated by summing the scores of all items (59).

##### 2.2.5.4. Thought control ability questionnaire

The *TCAQ* examines one’s capability to control negative thoughts. The questionnaire consists of 20 items, for example “It is easy for me to free myself of troublesome thoughts.” Each item is rated on a 5-point scale from 1 “strongly disagree” to 5 “strongly agree.” The final score is generated by summing the scores of all items (60).

##### 2.2.5.5. Thought suppression inventory

The *TSI* measures one’s ability to suppress unwanted thoughts. The inventory consists of 18 items, for example “I have thoughts which I would rather not have.” Each item is rated on a 5-point scale from 1 “strongly disagree” to 5 “strongly agree.” The full scale was administered, but items assessing one’s attempt at suppressing thoughts were excluded from data analysis as we were only interested in assessing the presence (or not) of negative thoughts. Overall, the effective suppression components and intrusion components were the only items included in the final analyses. The final score is generated by summing the scores of all items (61, 62).

##### 2.2.5.6. Ruminative response scale–brooding

The *RRS* examines features of ruminative thinking. The full questionnaire consists of 10 items and can be separated into two 5-item subscales (i.e., reflection, brooding), for example “think about a recent situation, wishing it had gone better.” Each item is rated on a 4-point scale from 1 “almost never” to 4 “almost always.” The final score is generated by summing the scores of all items. Participants were administered the entire scale, but only the brooding subscale (*RRS-B*) was included in data analysis, in that the brooding subscale focuses on negative thinking, while the reflection subscale better assesses adaptive features, such as coping with rumination (63).

The Functional Impairment Questions from the *Current Symptoms Checklist Scale* (64) and the *Adult Dispositional Hyperfocus Scale* (65) scales were administered to participants but not included in data analysis.

### 2.3. Procedure

All study procedures, across the three samples, were conducted online. Participants were provided a *Qualtrics* website link where they first completed informed consent. Then participants were presented with the questionnaires of the study assessing self-reported distraction (i.e., external distraction, spontaneous mind-wandering and unwanted intrusive thoughts) and ADHD symptomatology. Questionnaires were presented in a pseudo-random order such that questionnaires were categorized into 4 categories pertaining to: (1) external distraction, (2) spontaneous mind-wandering, (3) unwanted intrusive thoughts and (4) ADHD symptoms. These 4 categories were presented in a randomized order. Also, the order of the individual questionnaires within each category was randomized. However, in the ADHD category, the functional impairment questions are based on an individual’s current ADHD symptoms. Therefore, the functional impairment questions were always presented directly after the *ASRS* in that the *ASRS* assesses ADHD symptomatology.

After answering all questionnaires, participants provided their demographic information. Study procedures for the two large non-clinically evaluated samples took approximately 30 min to complete. Additionally, the clinically evaluated sample participated in a formal clinical interview using the *SCID-5-RV* to assess diagnosed ADHD, which was always completed after the online questionnaires *via* a password-protected Zoom meeting. Study procedures in the clinically evaluated sample took approximately 1 h. After all study procedures were completed, participants were debriefed and rewarded subject pool credit or compensated based on the respective sample to which they belonged.

### 2.4. Data analysis

Data analysis was conducted in the R (Version 4.0.3) environment. Scale items were reverse coded as necessary, so that higher scores always indicate higher levels of distraction. Normality was found to be satisfactory for all scale variables in all three samples.

The current study administered multiple scales for each distraction type. Because the primary focus of the current study is individual ADHD symptoms, for the clarity of the results we created composite scores to represent each distractor type (spontaneous mind-wandering, external distraction and unwanted intrusive thoughts) by averaging the z-scores of scales under each category^1^. Multicollinearity was found to be unsubstantial for the standardized distraction composite scores (spontaneous mind-wandering, external distraction and unwanted intrusive thoughts) in all three samples based on variance inflation factor scores (less than 10) and tolerance scores (greater than 0.2; see Supplementary material).

Dominance analyses were conducted using the *dominance analysis* package. For each dominance analysis, the outcome variables were the respective ADHD symptom and the predictor variables were the three types of distraction. To evaluate the robustness of the results, we performed each dominance analysis on 1,000 bootstrapped samples using the *bootdominanceanalysis* function (from the *dominance analysis* package). We examined the proportion of iterations that successfully replicate the original results (66).

Network analyses were conducted using the *bootnet* package and the *qgraph* package (67, 68). Specifically, we: (1) estimated Gaussian Graphical Model (GGM) psychological networks of ADHD symptoms and distraction scores which utilized graphical LASSO regularization and Extended Bayesian Information Criteria (EBIC) model selection for both non-clinically evaluated samples (EBIC tuning parameter = 0.5), (2) assessed the robustness and accuracy of the estimated network structures, (3) examined the centrality indices of the model, and (4) examined the edge weight differences between ADHD symptom nodes connected to distraction construct nodes. Edges in the network represent the regularized partial correlation between two specified nodes. We used regularized partial correlations in our network because partial correlations of zero (no association) are eliminated from the network model (44). Ultimately, this removes any potential spurious relationships between nodes and allows for a more interpretable network (44).

## 3. Results

Because there are three samples in our study, and because we conducted our analyses at the individual symptom level, there are multiple results to present. There are remarkably many commonalities among the analyses across all three samples. We summarize these commonalities before turning to the specifics. Through dominance analyses, we found mind-wandering was associated with the majority of the 18 ADHD symptoms. We found these effects to be stable and robust across symptoms and samples. When using network analysis, the significant differences that did emerge are consistent with our dominance analysis findings.

### 3.1. Descriptive statistics

Table 1 includes the descriptive statistics as indicated by the mean and standard deviations of the 18 ADHD symptoms for all three samples. All symptom items appear normal. However, see Supplementary material for full outline and descriptions of descriptive statistics. Additionally, all of the other scales measuring dimensions of distraction also appear normal with cronbach alphas of at least 0.6, which suggests all scales are satisfactorily reliable in measuring their intended constructs [except for the Thought Suppression Inventory (*TSI*) in the University Sample, which produced an alpha of 0.59; see Supplementary material].

**Table 1:** Descriptive statistics.

	Prolific sample (*N* = 651)	University sample (*N* = 569)	Clinically evaluated sample (*N* = 69)
Symptom	*M* (SD)	*M* (SD)	*M* (SD)
ASRS 1 | SCID 7 (Careless Mistakes)	1.77 (1.09)	1.74 (1.07)	0.32 (0.47)
ASRS 2 | SCID 8 (Diff. Sustaining Attention)	1.68 (1.06)	1.47 (1)	0.54 (0.5)
ASRS 3 | SCID 9 (Not Listening)	1.39 (1.09)	1.56 (1.1)	0.33 (0.47)
ASRS 4 | SCID 1 (Poor Follow Through)	2.13 (1.17)	2.52 (1.08)	0.26 (0.44)
ASRS 5 | SCID 2 (Diff. Organizing)	2.15 (1.33)	2.65 (1.2)	0.54 (0.5)
ASRS 6 | SCID 4 (Task Avoidance)	1.61 (1.13)	1.95 (1.05)	0.42 (0.5)
ASRS 7 | SCID 10 (Misplace Things)	1.62 (1)	1.99 (0.95)	0.57 (0.5)
ASRS 8 | SCID 11 (Distracted Extraneous Stimuli)	2.15 (1.12)	2.66 (0.97)	0.16 (0.37)
ASRS 9 | SCID 3 (Forgetful)	1.64 (1.15)	1.7 (1.04)	0.25 (0.43)
ASRS 10 | SCID 5 (Often Fidgets)	1.71 (1.16)	1.9 (1.07)	0.41 (0.49)
ASRS 11 | SCID 12 (Often Leaves Seat)	1.92 (1.07)	2.27 (0.95)	0.38 (0.49)
ASRS 12 | SCID 13 (Physically Restless)	0.89 (1.03)	1.01 (1)	0.15 (0.36)
ASRS 13 | SCID 14 (Diff. Relaxing/Unable Quietly)	1.95 (1.13)	2.16 (1.07)	0.37 (0.49)
ASRS 14 | SCID 6 (Drive by Motor)	1.86 (1.19)	1.88 (1.19)	0.54 (0.5)
ASRS 15 | SCID 15 (Talks Excessively)	1.33 (1.13)	1.79 (1.15)	0.28 (0.45)
ASRS 16 | SCID 16 (Blurts Out)	1.27 (1.14)	1.56 (1.19)	0.25 (0.44)
ASRS 17 | SCID 17 (Trouble Waiting)	1.12 (1.08)	1.34 (1.1)	0.18 (0.38)
ASRS 18 | SCID 18 (Interrupts Others)	1.19 (0.97)	1.43 (0.99)	0.28 (0.45)

### 3.2. Dominance analysis results

**Figure 1:**
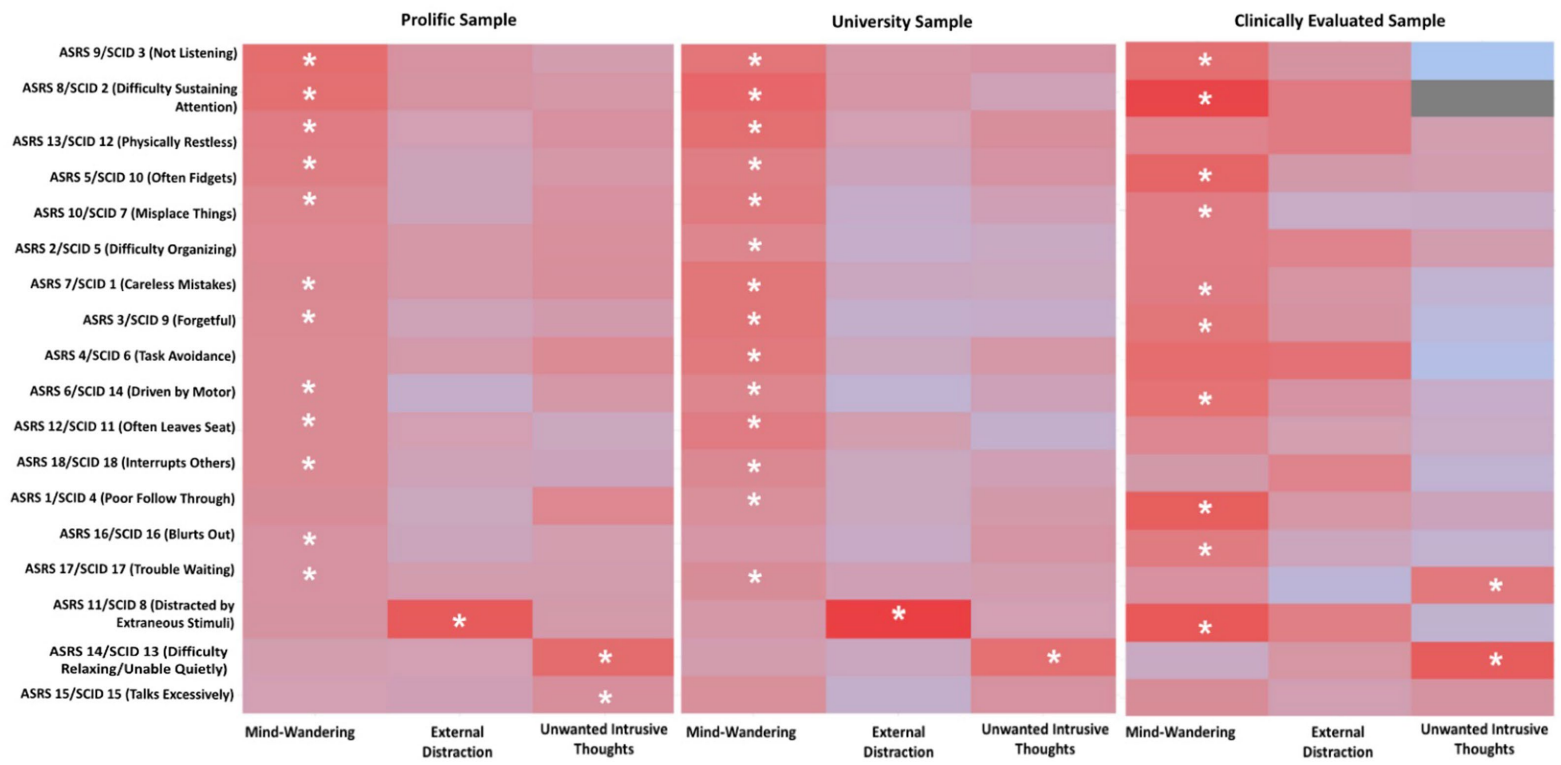
Heat Maps of Dominance Analysis Results. This figure demonstrates a combination of the results from multiple regression paired with dominance analysis. Regarding the multiple regression results, each row showcases the semi partial correlations between each distraction type (*x*-axis: EXT, External Distraction; MW, Mind-Wandering; UIT, Unwanted Intrusive Thoughts) and individual ADHD symptoms as indicated by the ASRS (Prolific and University Samples) and SCID [Clinically Evaluated Sample (*y*-axis)]. The more sharply shaded in red the cell is, the more variance that distraction type is explaining for the corresponding ADHD symptom. An asterisk is placed on the cell of the distraction type if that distraction type provided a significant explanation of variance in the multiple regression and completely dominated the other two types of distraction at least 70% of the time when bootstrapped at 1,000 iterations.

We conducted a dominance analysis for each of the 18 ADHD symptoms where predictors consisted of composite scores of spontaneous mind-wandering, external distraction and unwanted intrusive thoughts. The results of these dominance analyses are shown in Figure 1, where an asterisk denotes the distraction type that: (1) provides significant contribution of variance explained in the outcome ADHD symptom and (2) exerts *complete dominance* simultaneously over the two other types of distraction (with a replication rate of at least 70% when bootstrapped at 1,000 iterations). As an example, spontaneous mind-wandering completely dominates both external distraction and unwanted intrusive thoughts for the symptom *difficulty sustaining attention* (*ASRS* 8/*SCID* 2) in all three samples.

As shown in Figure 1, we found spontaneous mind-wandering to completely dominate both external distraction and unwanted intrusive thoughts for many of the ADHD symptoms across all three samples. It is noteworthy that spontaneous mind-wandering was a dominant predictor of several items associated with the inattention aspect of ADHD (e.g., *difficulty sustaining attention*) as well as several items associated with the hyperactivity/impulsivity aspect of ADHD (e.g., *often fidgets*).

While the dominance pattern of spontaneous mind-wandering was largely consistent across samples, several discrepancies can be found when comparing the results of the clinically elevated sample against those of the two larger samples. First, mind-wandering was the dominant predictor of *physically restless* (*ASRS* 13/*SCID* 12), *interrupting others* (*ASRS* 18/*SCID* 18), and *trouble waiting* (*ASRS* 17/*SCID* 17) in the two larger samples but not in the clinically evaluated sample. Second, mind-wandering was the dominant predictor of *distracted by extraneous stimuli* (*ASRS* 11/*SCID* 8) in the clinically evaluated sample but not in the two larger samples. Additionally, we find these results to be especially robust in that the replication rate ranges from 70 to 100% in most of the bootstrapped dominance analyses across all samples.

External distraction and unwanted intrusive thoughts also showcase instances of complete dominance. Across both non-clinically evaluated samples, unwanted intrusive thoughts completely dominated external distraction (Prolific Sample replication rate = 100%; University Sample replication rate = 100%) and spontaneous mind-wandering (Prolific Sample replication rate = 100%; University Sample replication rate = 99.7%) for the symptom *difficulty relaxing* (*ASRS*14). Lastly, external distraction completely dominated both spontaneous mind-wandering and unwanted intrusive thoughts for the symptom *distracted by extraneous stimuli* (*ASRS* 11/*SCID* 8) with replication rates of 100% in both non-clinically evaluated samples.

In the clinically evaluated sample, unwanted intrusive thoughts completely dominated external distraction and spontaneous mind-wandering for the symptoms *inability to do things quietly* (*SCID* 13) (external distraction replication rate = 98.2%; spontaneous mind-wandering replication rate = 99.0%) and *trouble waiting* (*ASRS* 17/*SCID* 17) (external distraction replication rate = 94.2%; spontaneous mind-wandering replication rate = 78.9%). Interestingly, *physically restless* (*ASRS* 13/*SCID* 12) showcases similar levels of significant contribution from both mind-wandering and external distraction, and both types of distraction completely dominate unwanted intrusive thoughts. However, we only denote an asterisk in Figure 1 if a distraction type simultaneously completely dominates the other two forms of distraction and provides a significant contribution in explaining the ADHD symptom. Surprisingly, no significant contribution is noted from any distraction type when explaining: *often leaves seat* (*ASRS* 12/*SCID* 11)*, talks excessively* (*ASRS* 15/*SCID* 15) *and interrupts others* (*ASRS* 18/*SCID* 18) in the clinically evaluated sample. Therefore, interpreting the dominance results is not necessary for these symptoms in this sample.

Overall, these dominance analysis results, across all three samples, support the notion that mind-wandering, overwhelmingly, is more closely associated with individual symptoms of ADHD. Ultimately, these results reveal a robust sense of heterogeneity among symptoms of ADHD in how they relate to different forms of distraction (see Supplementary materials for full results of each dominance analysis).

### 3.3. Network analyses

#### 3.3.1. Estimating networks

**Figure 2:**
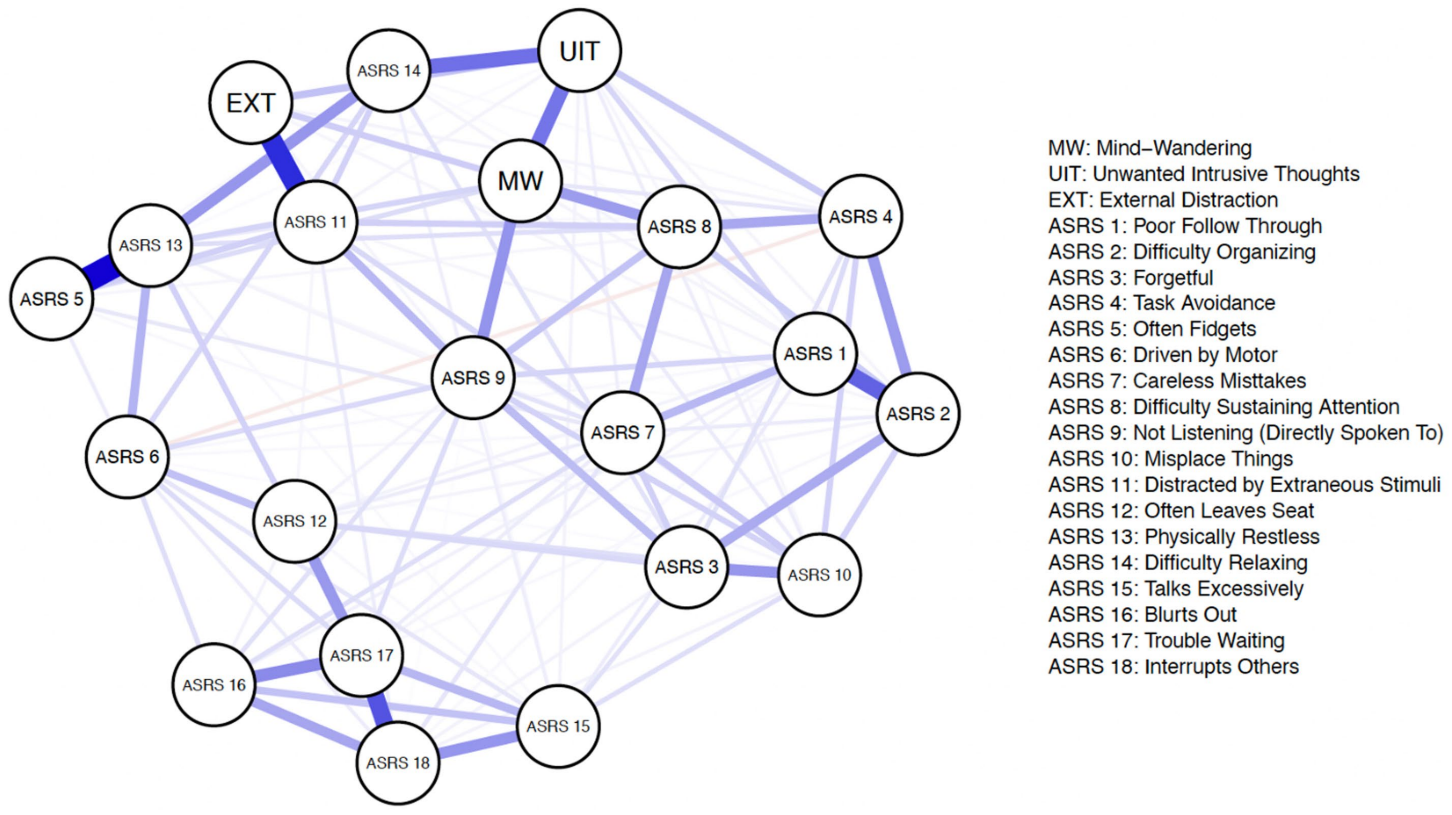
Estimated psychological network–prolific sample.

**Figure 3:**
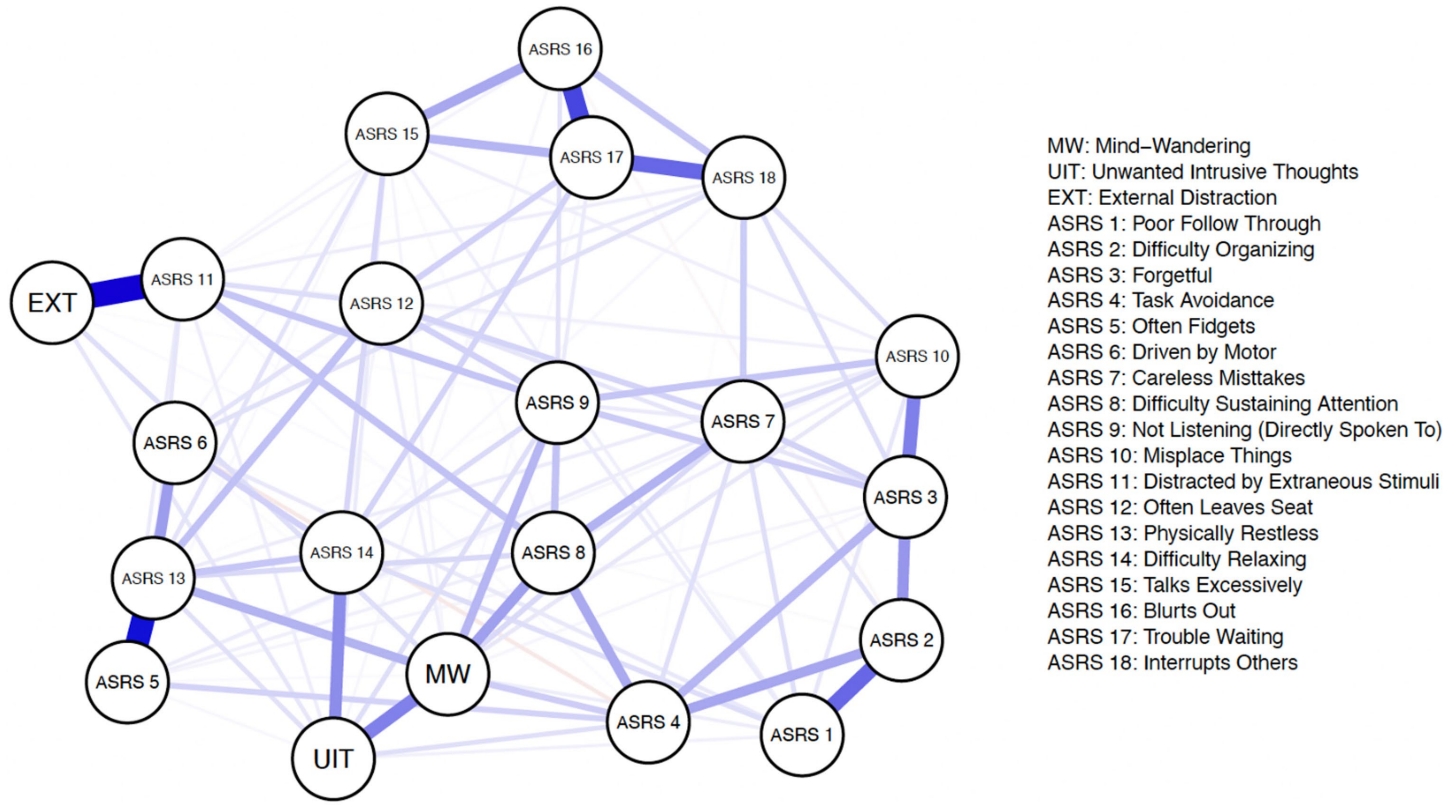
Estimated psychological network–university sample.

Figures 2, 3 illustrate the respective estimated Gaussian Graphical Model (GGM) psychological networks which utilized graphical LASSO regularization and Extended Bayesian Information Criteria (EBIC) model selection for both non-clinically evaluated samples. Each variable is represented as an individual node (circle) on the network plots. Each network displays 21 nodes composed of the 18 ADHD symptoms (*ASRS* items) and 3 dimensions of distraction (derived from composite scores from the respective distraction questionnaires).

#### 3.3.2. Node strength

First, we examined the network’s topological structure by computing node centrality indices. Of note, Epskamp et al. (42) recommended that prior to interpreting the centrality indices, the stability of these indices should be evaluated first to ensure the accuracy and robustness. We thus evaluated the stability of the indices by using the procedure described in Epskamp et al. (42). Specifically, we utilized the case-dropping subset bootstrap technique. We performed this procedure for both *strength*, *closeness*, and *betweenness*. The results, which can be found in the Supplementary material, indicate that only *strength* retained a satisfactory level of stability. Thus, we opted to report the results for *strength* in the main text and those for *closeness* and *betweenness* in the Supplementary material. *Strength* indicates how strongly a given variable is conditionally associated with other variables in the network. It is computed summing up the absolute edge weights for each node. A higher *strength* value, therefore, indicates that the variable has overall a stronger connection with other variables in the network.

**Figure 4:**
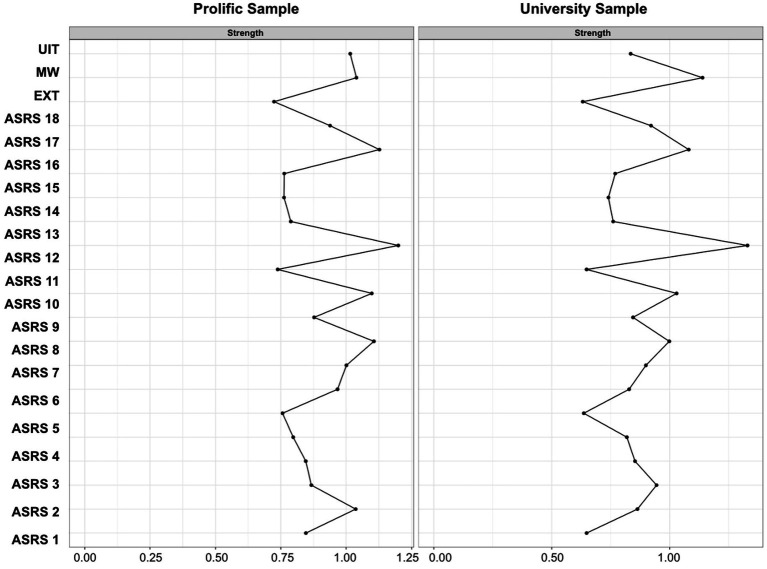
Network Strength Indices. This figure demonstrates the Strength indicator for all 21 variables included in the estimated networks for both the Prolific and University samples. EXT, External Distraction; MW, Mind-Wandering; UIT, Unwanted Intrusive Thoughts; ASRS, Adult ADHD Self-Report Rating Scale.

The results for *strength* are shown in Figure 4. *Physically restless* (*ASRS* 13) appears to be the most central node in both the Prolific and University samples. Of the distraction types, spontaneous mind-wandering (MW) appears to be the most central node across both samples. We further examined this intuition by conducting bootstrapped difference tests using the procedure described in Epskamp et al. (42). Across both samples we found the spontaneous mind-wandering node to have a significantly higher strength value compared to external distraction (EXT) (Prolific Sample: 95% CI [−0.44, −0.13]; University Sample: 95% CI [−0.66, −0.27]). Additionally, in the Prolific sample we found the unwanted intrusive thoughts (UIT) node to have significantly higher strength compared to external distraction (Prolific Sample: 95% CI [−0.42, −0.11]). And specific to the University sample, we found the spontaneous mind-wandering node to have significantly higher strength compared to unwanted intrusive thoughts in the University sample (University Sample: 95% CI [−0.52, −0.067]).

#### 3.3.3. Edge weight differences

Our research question can be further examined by assessing edge-weight differences. For example, we can assess if the edge weight between an *ASRS* item and one dimension of distraction (ex: *ASRS* 8–MW) is significantly different from the edge weight between the exact same *ASRS* item edge and another dimension of distraction (example: *ASRS* 8–EXT).

Prior to assessing edge-weight differences, we examined the accuracy of the edge-weights (42). In short, we generated bootsrapped 95% confidence intervals (CIs) of the edge weights. The edge weight CIs did not appear to completely overlap for all edges, which suggests some edges do significantly differ in connection strength. These results suggest our estimated network edge-weights appear visually accurate in that thicker nodes are depicting accurately stronger connections. Given that the edge-weights of relevant nodes appears to have satisfactory accuracies, we proceeded to compare the differences between these edge-weights. Further details regarding edge-weight accuracy can be found in the Supplementary material.

**Figure 5:**
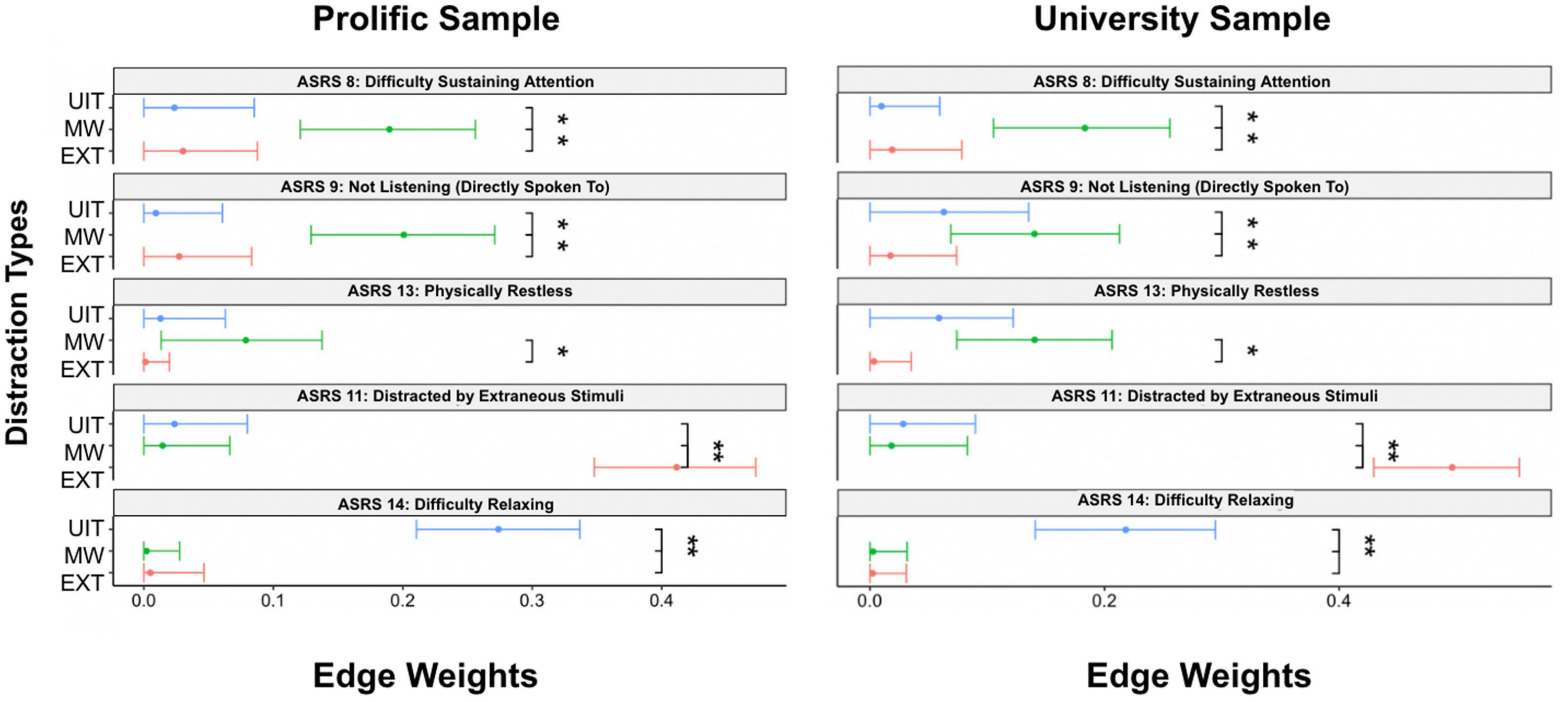
Edge Weight Differences. This figure demonstrates the ADHD symptoms in the estimated networks where significant edge weight differences emerged. EXT, External Distraction; MW, Mind-Wandering; UIT, Unwanted Intrusive Thoughts.

We compared the edge-weights by examining if the confidence intervals of the two edge-weights overlap. Non-overlapping confidence intervals indicate a significant difference between the edge-weights. Figure 5 displays the edge weight confidence intervals for the ADHD symptoms that showcase significant edge weight differences. It is important to first note that with using the network analysis approach, generally we notice fewer symptoms of ADHD that emerge indicating significant differences in relation to different types of distraction, this is likely due to the fact that network analysis inherently accounts for every inter-correlation within the network.

*ASRS* item 8 *(difficulty sustaining attention)* showcases a significantly stronger association to spontaneous mind-wandering (compared to external distraction and unwanted intrusive thoughts) across both the Prolific and University samples. *ASRS* item 9 *(not listening when directly spoken to*) showcases a significantly stronger association to spontaneous mind-wandering (compared to external distraction and unwanted intrusive thoughts) in the Prolific sample. However, in the University sample, *ASRS* item 9 *(not listening when directly spoken to*) only showcases a significantly stronger association to spontaneous mind-wandering when compared to external distraction (but not unwanted intrusive thoughts). *ASRS* item 13 (*physically restless*) reveals a significantly stronger association to spontaneous mind-wandering only when compared to external distraction (but not unwanted intrusive thoughts). Across both the Prolific and University samples, we find *ASRS* item 11 (*distracted by extraneous stimuli*) to have a significantly stronger connection to external distraction (compared to spontaneous mind-wandering and unwanted intrusive thoughts). Along similar lines across both samples, we find *ASRS* item 14 (*difficulty relaxing*) to have a significantly stronger association with unwanted intrusive thoughts (compared to spontaneous mind-wandering and external distraction).^2^ The results further support a sense of heterogeneity among ADHD symptoms in relation to different forms of distraction.

## 4. Discussion

In the present study, we tested whether the 18 symptoms of ADHD are differentially related to spontaneous mind-wandering, external distraction and unwanted intrusive thoughts in one clinically evaluated sample and two large non-clinically evaluated cohorts. Participants self-reported their susceptibility to external distraction, spontaneous mind-wandering, unwanted intrusive thoughts and ADHD symptoms. We utilized a combination of dominance analysis and network analysis to identify any symptoms that are not equally associated with each type of distraction, but rather are more related to a specific type of distraction while simultaneously accounting for the other forms of distraction.

Through dominance analysis, we found spontaneous mind-wandering to be especially important in explaining the majority of ADHD symptoms. It is noteworthy that spontaneous mind-wandering was a dominant predictor of several items associated with the inattention aspect of ADHD (e.g., difficulty *sustaining attention*) as well as several items associated with the hyperactivity/impulsivity aspect of ADHD (e.g., *often fidgets*). These results appear to support previous notions that spontaneous mind-wandering is strongly associated with both inattentive and hyperactive symptoms of ADHD (69).

We also note that some symptoms appear to be better explained by other forms of distraction besides spontaneous mind-wandering. For example, external distraction appears to best explain the symptom *distracted by extraneous stimuli* in the non-clinically evaluated samples, but not in the clinically evaluated sample. Additionally, unwanted intrusive thoughts best explained the symptom *difficulty relaxing* in our non-clinically evaluated samples, and *inability to do things quietly* in our clinically evaluated sample. These results seem plausible in that you might imagine someone who reports having issues relaxing or having issues with doing things in silence during idle time may experience negative recurring thoughts that interfere with calming their mind to relax or be comfortable during idle time filled with silence. Overall, the results of dominance analysis highlight there is something particularly interesting about the relationship between spontaneous mind-wandering and many symptoms of ADHD.

Next, we implemented network analyses. Specifically, we found *difficulty sustaining attention*, *not listening*, and *physically restless* to have significantly stronger associations to spontaneous mind-wandering compared to other sources of distraction. Consistent with our dominance analysis results, *distracted by extraneous stimuli* was significantly more related to composite external distraction, and *difficulty relaxing* was significantly more associated with composite unwanted intrusive thoughts.

As mentioned earlier, with network analysis all intercorrelations among variables in the estimated network are taken into account, which may explain why fewer symptoms showcase differential associations among the forms of distraction (compared to dominance analysis). This also allows for exploration of potential causal mechanisms within the network. For instance, *difficulty sustaining attention (ASRS 8)* may serve as a mediator between spontaneous mind-wandering (MW) and many *ASRS* items, such as *careless mistakes* (*ASRS* 7), *task avoidance* (*ASRS* 4) and *poor follow through* (*ASRS* 1) to name a few. This could potentially explain why in the dominance analysis results, these individual symptoms of *careless mistakes* (*ASRS* 7), *task avoidance* (*ASRS* 4) and *poor follow through* (*ASRS* 1) all showcase spontaneous mind-wandering as completely dominating the other forms of distraction. But in actuality, these symptoms may only be related to spontaneous mind-wandering indirectly, through *difficulty sustaining attention (ASRS 8)*. In other words, when *difficulty sustaining attention (ASRS 8)* is included simultaneously in a model with particular symptoms, such as *careless mistakes* (*ASRS* 7), the direct relationship between these particular symptoms and spontaneous mind-wandering disappears. Other potential mediators include: *not listening (directly spoken to) (ASRS 9), distracted by extraneous stimuli (ASRS 11),* and *difficulty relaxing (ASRS 14)* to name a few. It is important to note that these data are cross-sectional, so we are not suggesting any concrete causal claims at this point, but rather offering speculation as to why fewer symptoms indicate differential association to the forms of distraction assessed when using network analysis compared to dominance analysis.

Our results add to the small but growing body of literature suggesting that spontaneous mind-wandering is highly associated with ADHD (15, 18, 19). Indeed, our findings further support the argument that mind-wandering may be the defining feature of adult-ADHD, perhaps because of dysregulation of the default mode network (70–74). A review by Bozhilova et al. (71), proposes the *mind-wandering hypothesis*, which suggests irregular default mode network (DMN) brain activity in individuals with ADHD best explains the occurrence of heightened spontaneous mind-wandering. The DMN refers to a system of brain regions that are generally active during resting state activities in which an individual is not focused on carrying out a goal-directed activity, and mind-wandering is such a state (75–77). Specifically, the posterior cingulate cortex (PCC) and anterior medial prefrontal cortex (aMPFC) make up the core system within the DMN with additional subsystems, such as the dorsal medial prefrontal cortex (dMPFC) and medial temporal lobe (MTL) (78).

During goal-directed tasks, low frequency oscillations in the DMN are thought to weaken to allow for the necessary cognitive processes to unfold to successfully complete the task (79). However, the literature indicates that individuals with ADHD struggle with deactivating the DMN in that they experience continued low frequency oscillations when attempting to complete goal-directed tasks, which ultimately disrupts task performance (79–83). Taking together these findings, Bozhilova et al. (71) posits irregular deactivation capabilities of the DMN allows individuals with ADHD to remain in this resting state, default mode which gives way to abnormal levels of spontaneous mind-wandering. In turn the excessive mind-wandering presents inattentive ADHD symptoms and can lead to serious functional consequences in daily life.

We found it surprising that external distraction did not significantly explain more symptoms. The literature on ADHD is saturated with experimental studies that aim to understand distractibility within ADHD by using extraneous stimuli in the environment (e.g., noises, visual stimuli) (11–14). Being easily distracted by extraneous stimuli is thought to be an important symptom of ADHD in children (1)– with numerous scales only directly assessing external distraction and not internal distraction from mind-wandering (e.g., *ASRS* or *BAARS-IV*). With this emphasis on distraction from extraneous stimuli, we anticipated that external distraction would play a much larger role in explaining symptoms of ADHD. However, composite external distraction was only significantly more associated with the one symptom item that directly asked about being externally distracted in the non-clinically evaluated samples (*ASRS* 11: “how often are you distracted by activity or noise around you”), but not in the clinically evaluated sample (*SCID* 8: “...have you been very easily distracted by things going on around you that most others would have easily ignored like a car honking or other people talking?”). Perhaps this idiosyncrasy has to do with the difference in sample size. Our clinically, diagnosed sample includes only 69 individuals while our non-clinically evaluated samples both include over 500 individuals. Therefore, in future studies it would be beneficial to recruit a larger sample of clinically evaluated individuals if possible.

Lastly, we found unwanted intrusive thoughts to be significantly more associated with the symptom *difficulty relaxing* (*ASRS* 14) in both non-clinically evaluated samples and the symptom *inability to do things quietly* (*SCID* 13) in our clinically evaluated sample across multiple statistical methodologies. We find our results interesting for several reasons. First, we would like to discuss one inconsistency we noticed between one item from the *SCID-5-RV* and *ASRS* in terms of ADHD symptomatology. We used the gold-standard, *SCID-5-RV* to assess ADHD symptoms *via* clinical interview in our clinically evaluated sample (*N* = 69), but we used the *ASRS* questionnaire to assess self-reported ADHD symptomatology in our large non-clinically evaluated samples (N = 569, *N* = 651) in that no validated, participant-facing version of the *SCID-5-RV* is currently available for large-scale research studies. We noticed after data collection and analysis, that the *SCID-5-RV* directly assesses the symptom *inability to do things quietly* (*SCID* 13: “...have you often been unable to do something quietly in your spare time, like reading a book?”). However, the *ASRS* does not include any items on *inability to do things quietly*, rather this questionnaire includes an item that assesses *difficulty relaxing* (*ASRS* 14: “How often do you have difficulty unwinding and relaxing when you have time to yourself?”). When looking back at DSM-5 symptom criteria, only the *inability to do things quietly* (when free time arises) is a symptom of ADHD, but not *difficulty relaxing* (1). We are curious if this symptom was simply misinterpreted when developing this symptom item for the *ASRS.* Previous studies do note issues of low concordance between some *ASRS* items and DSM criteria of ADHD (53). We also assessed the concordance of corresponding symptoms of ADHD between *SCID-5-RV* items and *ASRS items* in our Clinically Evaluated sample, and also noted issues of low concordance on a couple of symptom items (see Supplementary material). Overall, this speaks to the need for future studies that focus on the accuracy and utility of ADHD screeners and assessment tools.

Looking past this inconsistency, Jonkman et al. (16) and Mitchell et al. (27) found a positive relationship between inattentive-ADHD symptoms and *intrusive rumination* and *negative automatic thoughts* (respectively), but not between hyperactive/impulsive-ADHD symptoms and *intrusive rumination* or *negative automatic thoughts*. In that *inability to do things quietly* (*SCID* 13) is considered a hyperactive/impulsive symptom of ADHD, it is intuitive to predict this symptom would not be related to unwanted intrusive thoughts based on these prior studies, but our study finds the opposite. Perhaps our results are different than expected because we directly examined the relationship between the symptom *inability to do things quietly* (*SCID* 13) and unwanted intrusive thoughts, while previous studies correlated higher order sub-symptom categories of inattentive-ADHD and hyperactive/impulsive-ADHD symptomatology with negative rumination. This further highlights the utility of the individual symptom approach in that this nuance may be overlooked when utilizing an aggregated approach.

We used the same datasets for our large non-clinically evaluated samples as Zhang et al., (26) (*N* = 569, *N* = 651). As stated previously, Zhang et al., utilized a common factor approach to understand distractibility in relation to ADHD symptomatology, and found general distractibility (extracted from mind-wandering, external distraction and unwanted intrusive thoughts) to better explain latent ADHD symptomatology. Our goal differed in that we wanted to assess whether specific symptoms of ADHD might be related to specific types of distraction and found that mind-wandering is closely tied to many other symptoms. As such, our conclusions are not inconsistent with the conclusions of Zhang et al. Additionally, these results are not meant to suggest that external distraction and unwanted intrusive thoughts are not relevant to ADHD. In fact, in our clinically evaluated sample we found those meeting criteria for ADHD (EXT: *M* = 0.44, *SD* = 0.64; UIT: *M* = 0.31, *SD* = 0.92) and those not currently meeting criteria for ADHD (controls) (EXT: *M* = −0.34, *SD* = 0.97; UIT: *M* = −0.24, *SD* = 0.77) significantly differed in scores of external distraction [*t*(65.8) = −4.01, *p* < 0.001)] and unwanted intrusive thoughts [*t*(55.9) = −2.66, *p* < 0.05) with large (*d* = −0.93) to medium (*d* = −0.66)] effect sizes, respectively, indicating that although mind-wandering emerged as the relatively more important form of distraction for many symptoms of ADHD, external distraction and unwanted intrusive thoughts appear to still be relevant for understanding ADHD.

### 4.1. Limitations

Our data are correlational, so we cannot definitively say whether or not a particular type of distraction causes certain symptoms, or whether those symptoms cause distraction. Another limitation is that all questionnaires were given in the same training session, so we cannot fully exclude the possibility of carryover effects such that responses on one questionnaire influenced another. The fact that the questionnaires were pseudo-randomized across participants reduces this concern. Lastly, we would like to acknowledge all data was collected online in 2020 in the midst of a global pandemic and political unrest in uncontrolled environments outside of the laboratory.

## 5. Conclusion

In conclusion, spontaneous mind-wandering appears to be especially important in explaining many individual symptoms of ADHD across clinically evaluated and non-clinically evaluated samples. Some symptoms do seem to be better explained by other forms of distraction, which ultimately suggests ADHD symptoms display a heterogeneous relationship with different forms of distraction. To our knowledge our study is the first of its kind to examine multiple forms of distraction to better understand ADHD symptomatology. Future research should utilize experimental tasks to induce these different forms of distraction to corroborate our findings in controlled experimental paradigms. Lastly, we hope our findings draw attention to the need for continued research centered around understanding the relationship between spontaneous mind-wandering and adult-ADHD.

## Data availability statement

The datasets presented in this study can be found in online repositories. The names of the repository/repositories and accession number(s) can be found at: https://osf.io/2ndgj/.

## Ethics statement

The studies involving human participants were reviewed and approved by the University of Michigan Health Sciences and Behavioral Sciences Institutional Review Board (IRB-HSBS). The patients/participants provided their written informed consent to participate in this study.

## Author contributions

JO and HZ: study design, study implementation, data analysis, writing original draft, and writing review and editing. MC: study implementation and writing review and editing. PS and JJ: study design, study implementation, data analysis, writing original draft, and writing review and editing. All authors contributed to the article and approved the submitted version.

## Funding

This work was supported in part by a grant from National Institute of Mental Health (Unique Federal Award Identification Number (FAIN): R21MH129909) to JJ.

## Conflict of interest

The authors declare that the research was conducted in the absence of any commercial or financial relationships that could be construed as a potential conflict of interest.

## Publisher’s note

All claims expressed in this article are solely those of the authors and do not necessarily represent those of their affiliated organizations, or those of the publisher, the editors and the reviewers. Any product that may be evaluated in this article, or claim that may be made by its manufacturer, is not guaranteed or endorsed by the publisher.

## Footnotes

^1^A confirmatory factor analysis of the 12 distraction scales using the two larger non-clinically evaluated samples (the Prolific sample and the University sample) obtained a good fit for both samples. Measures of external distraction, unwanted intrusive thoughts and mind-wandering formed three distinct and yet correlated factors with high loadings for each factor. See ﻿supplementary material﻿ for more details.

^2^One unique result emerged from the University sample when assessing edge weight differences of the estimated network. *ASRS* item 4 (*task avoidance*) showcased significantly stronger association with mind-wandering (compared to external distraction) (see ﻿Supplementary material﻿).
