# Supplementary material for: The association between different sources of distraction and symptoms of attention deficit hyperactivity disorder
Source: Front Psychiatry. 2023 Jul 27;14:1173989. doi: 10.3389/fpsyt.2023.1173989 (PMC10421702; doi:10.3389/fpsyt.2023.1173989)
Supplement: Supplementary file 1 [file Data_Sheet_1.DOCX]

Supplementary Material

The Association Between Different Sources of Distraction and Symptoms of Attention Deficit Hyperactivity Disorder

Jahla B. Osborne^*^, Han Zhang, Madison Carlson, Priti Shah, John Jonides

*** Correspondence:** Corresponding Author: [jahlao@umich.edu](mailto:jahlao@umich.edu)

# Assessing Separable Distraction Constructs with Confirmatory Factor Analysis

To ensure the distraction questionnaires were measuring separable constructs we performed confirmatory factor analysis on our two large non-clinically evaluated samples (see Figure 1). In the rectangles we have our observed variables which are our individual scales, and in the ellipses we have our latent distraction constructs of mind-wandering (MW), unwanted intrusive thoughts (UIT), and external distraction (EXT). Factor loadings loaded well onto their intended latent constructs. Additionally, model fit is acceptable per the model fit indices (*RMSEA* between 0.05 and 0.08 , *CFI* greater than .9) in both models. Overall, these models suggest external distraction, mind-wandering and unwanted intrusive thoughts are indeed related yet distinct constructs.

## Supplementary Figure 1 - *Confirmatory Factor Analyses*


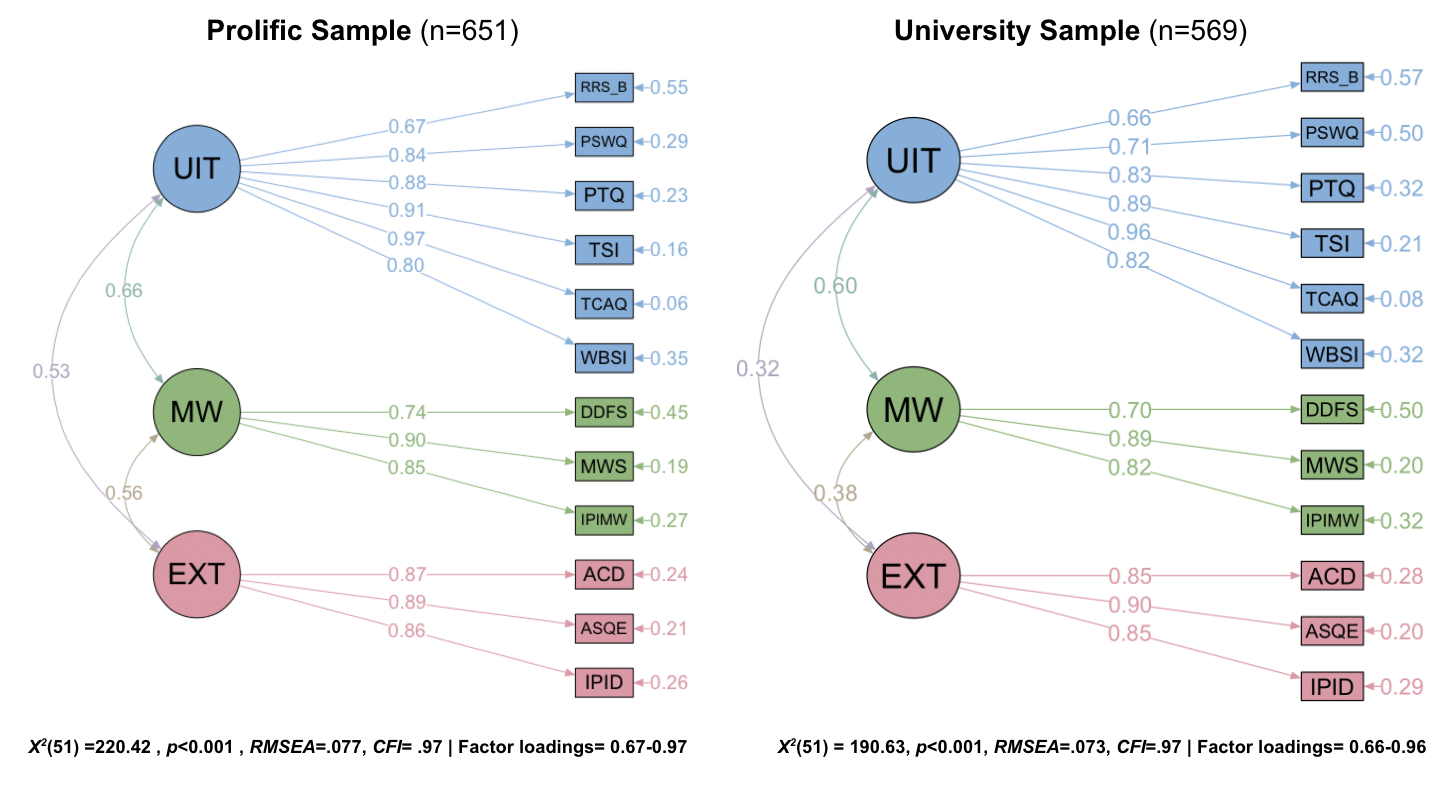


Note. MW= mind-wandering, EXT= external distraction, UIT= unwanted intrusive thoughts, IPIMW= Imaginal Processes Inventory - Mind-wandering, MWS= Mind-wandering – Spontaneous, DDFS= Daydreaming Frequency Scale, ACD=Attentional Control - Distraction; IPID= Imaginal Processes Inventory – Distractibility, ASQE= Attentional Style Questionnaire – External, PTQ= Pervasive Thinking Questionnaire, WBSI= White Bear Suppression Inventory, PSWQ=Penn State Worry Questionnaire,TCAQ= Thought Control Ability Questionnaire, TSI= Thought Suppression Inventory, RRS-B= Ruminative Response Scale- Brooding.

# Assessing Multicollinearity of Composite Distraction Scores

To assess the potential multicollinearity of our composite distraction types we calculated the variance inflation factor (VIF) and tolerance scores. VIF scores less than 10 and tolerance scores greater than 0.2 are considered acceptable (see Supplementary Table 1).

## Supplementary Table 1 *- Variance Inflation Factor & Tolerance Scores*

|  | **Prolific Sample** | | **University Sample** | | **Clinically Evaluated Sample** | |
| --- | --- | --- | --- | --- | --- | --- |
| **Distraction Type** | **VIF** | **Tolerance** | **VIF** | **Tolerance** | **VIF** | **Tolerance** |
| Mind-Wandering | 1.76 | 0.57 | 1.44 | 0.69 | 1.83 | 0.55 |
| External Distraction | 1.40 | 0.71 | 1.14 | 0.88 | 1.33 | 0.75 |
| Unwanted Intrusive Thoughts | 1.74 | 0.57 | 1.40 | 0.71 | 1.67 | 0.60 |

# Descriptive Statistics

Supplementary Table 2 includes the descriptive statistics of the 18 ADHD symptoms for all three samples. All continuous scale items from the ASRS in the large non-clinically evaluated samples appear normal, for skewness and kurtosis are within the +/- 2 acceptable range. We also provide descriptive statistics for the binary scale items from the SCID-5-RV in the clinically evaluated sample as well. It is important to note that since these items are binary they cannot be normally distributed. Therefore, there are instances where skewness and kurtosis scores violate the acceptable range criteria. We provide the descriptive statistics for all of the other scales measuring dimensions of distraction in our “Additional Supplementary Materials” file on Open Science Framework (OSF). These scales also appear normal with respect to skewness and kurtosis, and all cronbach alphas are at least 0.6, which suggests all scales are satisfactorily reliable in measuring their intended constructs (except for the Thought Suppression Inventory in the University Sample, which produced an alpha of 0.59).

**Link to Additional Supplementary Materials:**

<https://osf.io/2ndgj/files/osfstorage/63f9f557bbc5e50381f801f9>

**Supplementary Table 2** - *Descriptive Statistics (ASRS Items)*

|  | **Prolific Sample**  **(N = 651)** | | | **University Sample**  **(N = 569)** | | | **Clinically Evaluated Sample**  **(N = 69)** | | |
| --- | --- | --- | --- | --- | --- | --- | --- | --- | --- |
| **Construct** | ***M (SD)*** | ***Skew*** | ***Kurt*** | ***M(SD)*** | ***Skew*** | ***Kurt*** | ***M(SD)*** | ***Skew*** | ***Kurt*** |
| ASRS 1 \| SCID 7  (Careless Mistakes) | 1.77(1.09) | 0.24 | -0.69 | 1.74(1.07) | 0.52 | -0.44 | 0.32(0.47) | 0.76 | -1.44 |
| ASRS 2 \| SCID 8  (Diff. Sustaining Attention) | 1.68(1.06) | 0.3 | -0.55 | 1.47(1) | 0.64 | -0.01 | 0.54(0.5) | -0.17 | -2 |
| ASRS 3 \| SCID 9  (Not Listening) | 1.39(1.09) | 0.63 | -0.23 | 1.56(1.1) | 0.55 | -0.3 | 0.33(0.47) | 0.69 | -1.54 |
| ASRS 4 \| SCID 1  (Poor Follow Through) | 2.13(1.17) | -0.07 | -0.78 | 2.52(1.08) | -0.3 | -0.75 | 0.26(0.44) | 1.07 | -0.88 |
| ASRS 5 \| SCID 2  (Diff. Organizing) | 2.15(1.33) | -0.13 | -1.14 | 2.65(1.2) | -0.54 | -0.71 | 0.54(0.5) | -0.14 | -2.01 |
| ASRS 6 \| SCID 4  (Task Avoidance) | 1.61(1.13) | 0.32 | -0.67 | 1.95(1.05) | 0.09 | -0.59 | 0.42(0.5) | 0.32 | -1.93 |
| ASRS 7 \| SCID 10  (Misplace Things) | 1.62(1) | 0.4 | -0.27 | 1.99(0.95) | 0.35 | -0.26 | 0.57(0.5) | -0.29 | -1.94 |
| ASRS 8 \| SCID 11  (Distracted Extraneous Stimuli) | 2.15(1.12) | -0.11 | -0.67 | 2.66(0.97) | -0.27 | -0.58 | 0.16(0.37) | 1.8 | 1.25 |
| ASRS 9 \| SCID 3  (Forgetful) | 1.64(1.15) | 0.32 | -0.69 | 1.7(1.04) | 0.45 | -0.29 | 0.25(0.43) | 1.15 | -0.68 |
| ASRS 10 \| SCID 5  (Often Fidgets) | 1.71(1.16) | 0.37 | -0.68 | 1.9(1.07) | 0.3 | -0.65 | 0.41(0.49) | 0.38 | -1.89 |
| ASRS 11 \| SCID 12  (Often Leaves Seat) | 1.92(1.07) | 0.14 | -0.64 | 2.27(0.95) | -0.01 | -0.48 | 0.38(0.49) | 0.47 | -1.8 |
| ASRS 12 \| SCID 13  (Physically Restless) | 0.89(1.03) | 1.07 | 0.45 | 1.01(1) | 0.97 | 0.49 | 0.15(0.36) | 1.95 | 1.83 |
| ASRS 13 \| SCID 14  (Diff. Relaxing/Unable Quietly) | 1.95(1.13) | 0.17 | -0.71 | 2.16(1.07) | 0.09 | -0.63 | 0.37(0.49) | 0.54 | -1.74 |
| ASRS 14 \| SCID 6  (Drive by Motor) | 1.86(1.19) | 0.14 | -0.87 | 1.88(1.19) | 0.19 | -0.86 | 0.54(0.5) | -0.17 | -2 |
| ASRS 15 \| SCID 15  (Talks Excessively) | 1.33(1.13) | 0.51 | -0.62 | 1.79(1.15) | 0.39 | -0.61 | 0.28(0.45) | 0.96 | -1.09 |
| ASRS 16 \| SCID 16  (Blurts Out) | 1.27(1.14) | 0.7 | -0.28 | 1.56(1.19) | 0.41 | -0.71 | 0.25(0.44) | 1.13 | -0.73 |
| ASRS 17 \| SCID 17  (Trouble Waiting) | 1.12(1.08) | 0.72 | -0.24 | 1.34(1.1) | 0.57 | -0.4 | 0.18(0.38) | 1.66 | 0.77 |
| ASRS 18 \| SCID 18  (Interrupts Others) | 1.19(0.97) | 0.75 | 0.31 | 1.43(0.99) | 0.62 | 0.09 | 0.28(0.45) | 0.96 | -1.09 |

Note. ASRS=Adult ADHD Self-report Scale, SCID=Structured Clinical Interview for DSM-5 Research Version.

# Multiple Regression & Dominance Analyses Results

We provide the full regression analysis output directly followed by the full dominance analysis outputs for each ADHD symptom regression by sample. We report the unstandardized beta weights (*b*) , standard error (*SE*), and t-value (*t*). The larger the unstandardized beta weight, the larger the contribution the predictor variable is contributing to the explained variance.

Dominance analysis is commonly paired with multiple regression to better understand the compartmentalization of the explained variance (R^2^). Dominance can be defined at varying levels ranging from *complete dominance* (highest level) to *general dominance* (lowest level) (Azen & Budescu, 2003; Azen & Budescu, 2006). To understand *complete dominance*, one must first understand the concept of additional contribution. Additional contribution occurs when the R^2^ increases after a predictor is included in a subset regression model that contains other predictor(s) (Azen & Budescu, 2003; Azen & Budescu, 2006). *Complete dominance* occurs when the additional contribution for one predictor is always larger than the additional contributions of other predictors in subset models (Azen & Budescu, 2003; Azen & Budescu, 2006). In our results’ tables we provide the initial dominance analysis result (*Dij*) as well as the reproducibility of this initial result when iterated 1,000 times (*rep*).

**Please visit our repository on Open Science Framework (OSF) for full table output results for multiple regression and dominance analyses under the file name “Additional Supplementary Material”. We provide these on Open Science Framework (OSF) due to the restrictions on the maximum number of tables and figures.**

**Link to Additional Supplementary Materials:**

<https://osf.io/2ndgj/files/osfstorage/63f9f557bbc5e50381f801f9>

# Network Accuracy

According to Epskamp et al., (2018), network accuracy is imperative when drawing conclusions based on estimated networks. Specifically, they posit after estimating a network both edge-weight accuracy and stability of centrality indices should be conducted. These analyses help to suggest whether the network is likely accurate or that the network visualization may be inaccurate or deceptive. We conducted these assessments prior to completing our main analysis of examining potential edge weight differences within the network.

## *Edge-Weight Accuracy*

Figure 2 illustrates the estimated edge-weights (red dots) and 95% CI (gray shaded area) (Epskamp et al., 2018). The Y-axis represents each edge in the network (Epskamp et al., 2018). The 95% CIs surrounding the edge-weights, in both non-clinically evaluated samples, appear to be narrow. Additionally, the edge-weight CIs only seem to overlap in the middle of the graph. The edge weight CIs do not appear to completely overlap for all edges, which suggests some edges do significantly differ in connection strength. These results suggest our estimated network edge-weights appear visually accurate in that thicker nodes are depicting accurately stronger connections.

Additionally, in the main text we only provided a figure of the edge weights where significant differences emerged that were consistent across both non-clinically evaluated samples (Prolific Sample, University Sample). In Figure 3A and Figure 3B we provide the edge weight difference results for all ASRS items regardless if significant differences emerged.

**Supplementary Figure 2** - *Edge Weight Accuracy Plot*


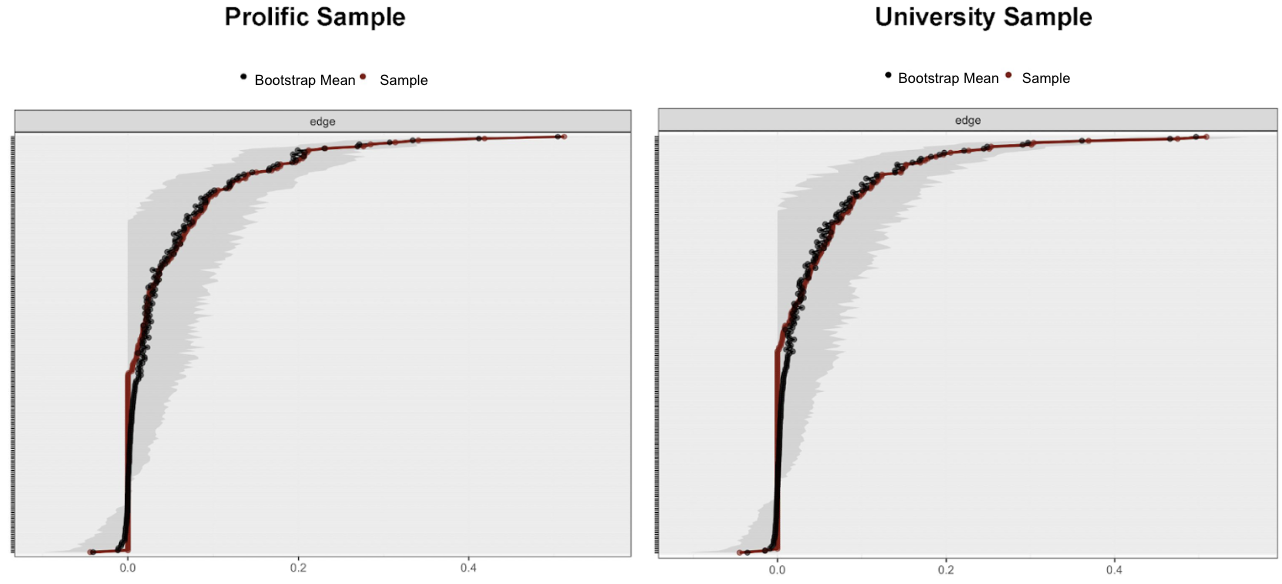


**Supplementary Figure 3A** - *Edge Weight Differences (Items 1-9)*

**
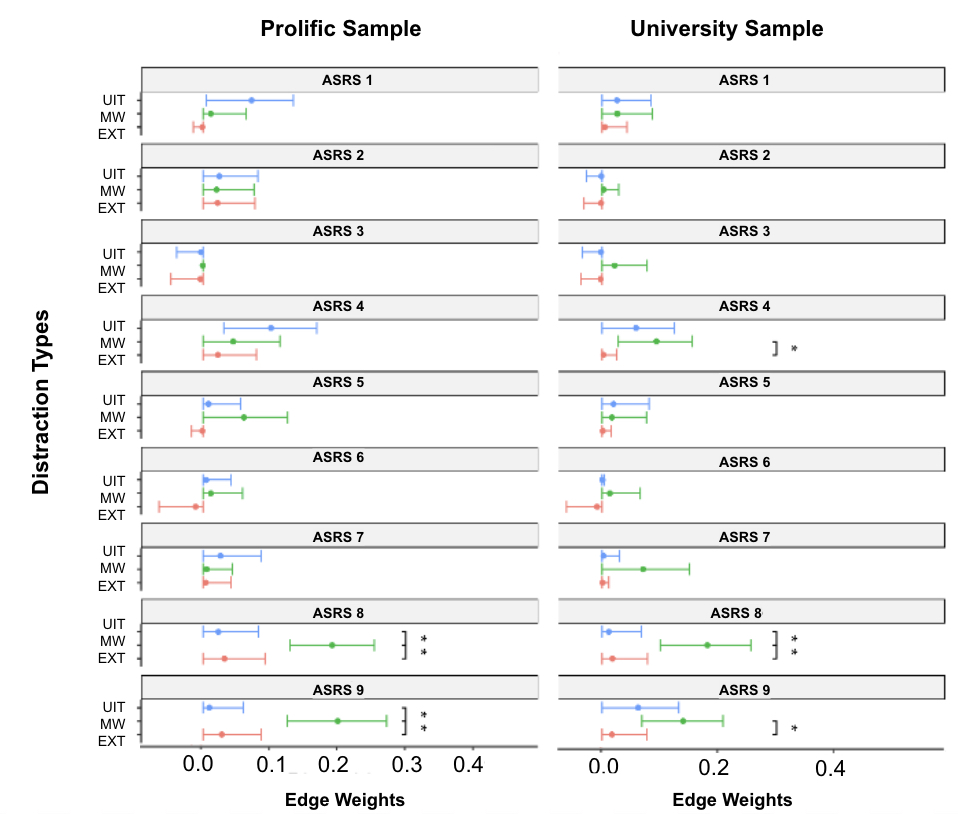
**

**Supplementary Figure 3B** - *Edge Weight Differences (Items 10-18)*

**
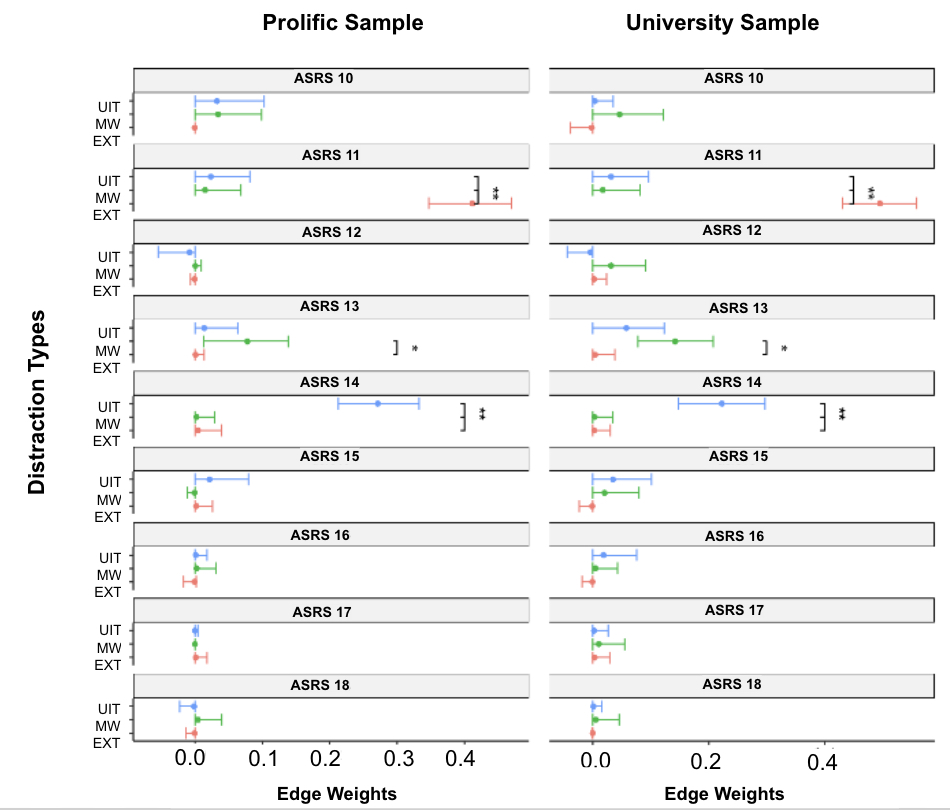
**

**5.2 *Stability of Centrality Indices***

Figure 4 showcases the *strength*, *betweenness*, and *closeness* centrality indices. *Strength* equates to the total number of edges that are directly connected to a node (Epskamp et al., 2017). More central nodes have more connections. ASRS-13 “physically restless” appears to be the most central node in both the prolific and university samples. *Closeness* measures the total number of edges that are indirectly connected to a node (Epskamp et al., 2017). *Not listening* (ASRS item 9) appears to have the highest indirect connections in the Prolific sample, while *difficulty sustaining attention* (ASRS item 8) appears to have the most indirect connections in the University sample. Of the distraction types, spontaneous mind-wandering (MW) appears to have the most indirect connections as well. *Betweenness* indicates which nodes are important for bridging relationships between two other nodes (Epskamp et al., 2017). *Not listening* (ASRS item 9) showcases the highest level of betweenness, suggesting it is the most important node for connecting other nodes in the prolific sample. However, physically restless (ASRS item 13) appears to have the highest level of betweenness in the university sample. Of the distraction types, spontaneous mind-wandering (MW) appears to have greater betweenness in the University sample, but unwanted intrusive thoughts appears to have greater betweenness in the Prolific sample.We utilized the *corStability* function from the *bootne*t package to assess the stability of these indices and discovered that the *strength* indice remains satisfactorily stable even when reducing the number cases included in the analysis (see Figure 5). However, *closeness* and *betweenness* do not show the same level of stability.

**Supplementary Figure 4** - *Network Centrality Indices*

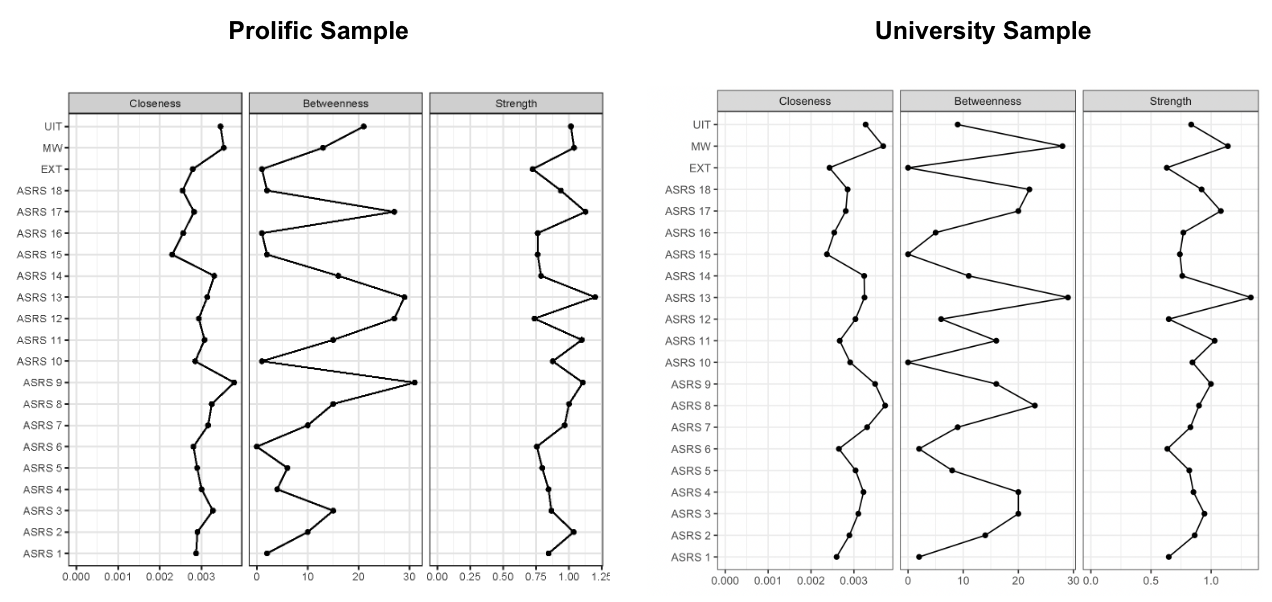


**Supplementary Figure 5** - *Stability of Centrality Indices*


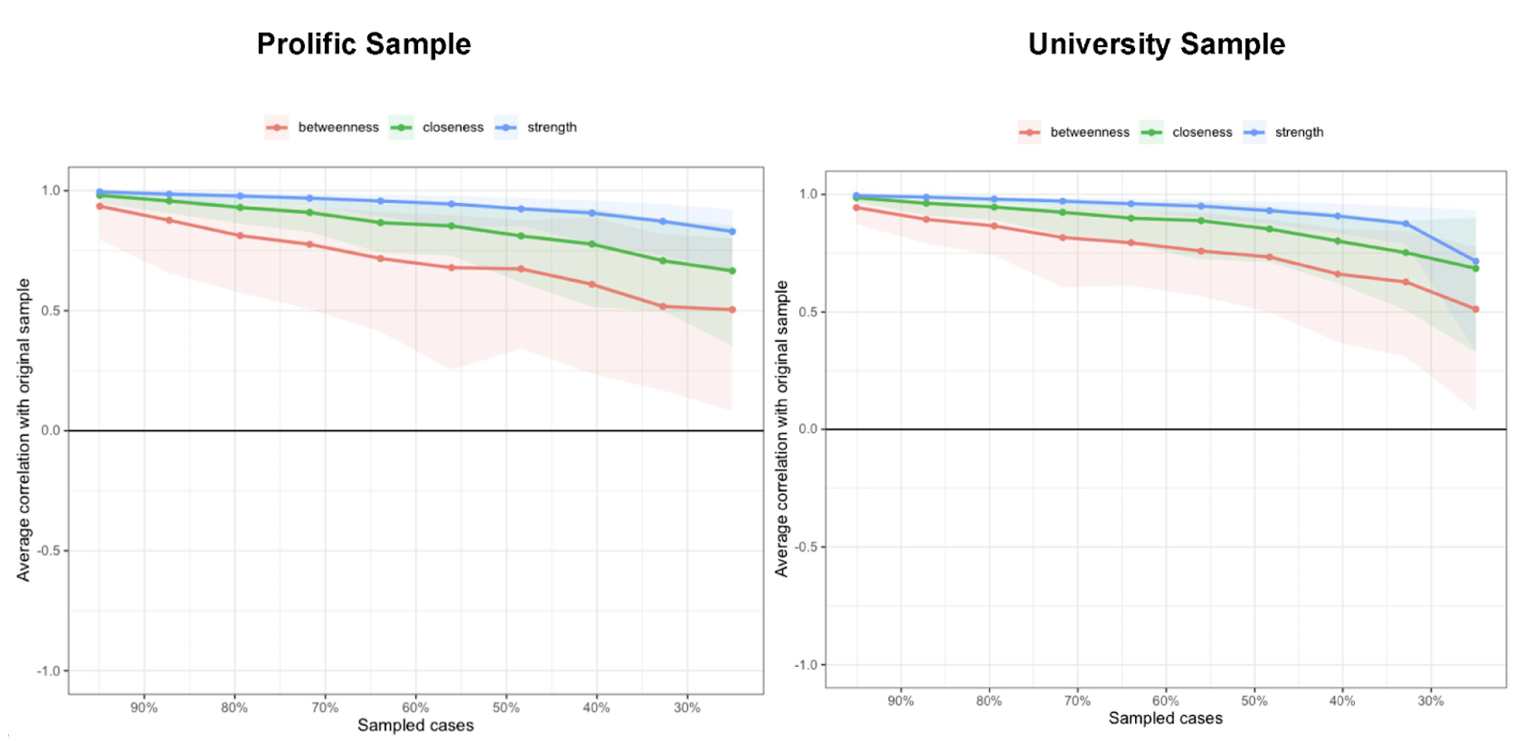


# Concordance Between ADHD Measures

We examined the concordance of the individual symptom items from our two measures of ADHD symptomatology (Adult ADHD Self Report Scale (ASRS), Structured Clinical Interview for DSM-5 Research Version (SCID-5-RV)) in our clinically evaluated sample. We were looking to see how well the ASRS items correlated with the respective “gold standard” item from the SCID-5-RV. All ASRS items were significantly associated with their clinical symptom item from the SCID-5-RV. However, concordance varied from r = 0.39 - 0.68 (see Supplementary Table 3).

**Supplementary Table 3** - *Concordance of ADHD Symptoms*

| **Construct** | **SCID Item** | **ASRS Item** | ***r*** | ***p*** |
| --- | --- | --- | --- | --- |
| Careless Mistakes | 1 | 7 | 0.45 | <0.001 |
| Difficulty Sustaining Attention | 2 | 8 | 0.59 | <0.001 |
| 1. Not Listening (Directly Spoken To) | 3 | 9 | 0.36 | <0.01 |
| 1. Poor Follow Through | 4 | 1 | 0.47 | <0.001 |
| 1. Difficulty Organizing | 5 | 2 | 0.44 | <0.001 |
| 1. Avoids Task | 6 | 4 | 0.50 | <0.001 |
| 1. Misplace Things | 7 | 10 | 0.60 | <0.001 |
| 1. Distracted (Externally) | 8 | 11 | 0.55 | <0.001 |
| 1. Forgetful | 9 | 3 | 0.45 | <0.001 |
| 1. Fidgets | 10 | 5 | 0.68 | <0.001 |
| 1. Leaves Seat | 11 | 12 | 0.51 | <0.001 |
| 1. Physically Restless | 12 | 13 | 0.51 | <0.001 |
| 1. Unable to do things quietly/Diff. relaxing | 13 | 14 | 0.39 | <0.001 |
| 1. Driven by Motor | 14 | 6 | 0.59 | <0.001 |
| 1. Talks Excessively | 15 | 15 | 0.65 | <0.001 |
| 1. Blurts Out | 16 | 16 | 0.49 | <0.001 |
| 1. Trouble Waiting | 17 | 17 | 0.40 | <0.001 |
| 1. Interrupts Others | 18 | 18 | 0.46 | <0.001 |
